# *Dehalococcoides mccartyi* strain CBDB1 takes up protons from the cytoplasm to reductively dehalogenate organohalides indicating a new modus of proton motive force generation

**DOI:** 10.3389/fmicb.2023.1305108

**Published:** 2023-12-22

**Authors:** Nadine Hellmold, Marie Eberwein, My Hanh Thi Phan, Steffen Kümmel, Oliver Einsle, Darja Deobald, Lorenz Adrian

**Affiliations:** ^1^Department Environmental Biotechnology, Helmholtz Centre for Environmental Research-UFZ, Leipzig, Germany; ^2^Institute of Biotechnology, Technische Universität Berlin, Berlin, Germany; ^3^Department Isotope Biogeochemistry, Helmholtz Centre for Environmental Research-UFZ, Leipzig, Germany; ^4^Institute of Biochemistry, Albert-Ludwigs-Universität Freiburg, Freiburg im Breisgau, Germany

**Keywords:** energy conservation, organohalide respiration (OHR), reductive dehalogenase, proton translocation, proton motive force *(pmf)*, proton channel, NrfD

## Abstract

Proton translocation across the cytoplasmic membrane is a vital process for all organisms. *Dehalococcoides* strains are strictly anaerobic organohalide respiring bacteria that lack quinones and cytochromes but express a large membrane-bound protein complex (OHR complex) proposed to generate a proton gradient. However, its functioning is unclear. By using a dehalogenase-based enzyme activity assay with deuterium-labelled water in various experimental designs, we obtained evidence that the halogen atom of the halogenated electron acceptor is substituted with a proton from the cytoplasm. This suggests that the protein complex couples exergonic electron flux through the periplasmic subunits of the OHR complex to the endergonic transport of protons from the cytoplasm across the cytoplasmic membrane against the proton gradient to the halogenated electron acceptor. Using computational tools, we located two proton-conducting half-channels in the AlphaFold2-predicted structure of the OmeB subunit of the OHR complex, converging in a highly conserved arginine residue that could play a proton gatekeeper role. The cytoplasmic proton half-channel in OmeB is connected to a putative proton-conducting path within the reductive dehalogenase subunit. Our results indicate that the reductive dehalogenase and its halogenated substrate serve as both electron and proton acceptors, providing insights into the proton translocation mechanism within the OHR complex and contributing to a better understanding of energy conservation in *D. mccartyi* strains. Our results reveal a very simple mode of energy conservation in anaerobic bacteria, showing that proton translocation coupled to periplasmic electron flow might have importance also in other microbial processes and biotechnological applications.

## 1 Introduction

A chemiosmotic potential across the cytoplasmic membrane is fundamental to all life forms. In prokaryotes, the chemiosmotic potential is primarily formed by a concentration gradient of protons or sodium ions across the cytoplasmic membrane thus a charge separation across the membrane. These gradients are predominantly generated by redox reaction-driven respiratory chains, resulting in a proton motive force (*pmf*). The *pmf* is the sum of electrostatic and osmotic potentials, typically measures around 150 mV (Schoepp-Cothenet et al., 2013) and serves as an energy source for many cellular processes, including ATP regeneration through membrane-bound ATPase, active transport, cellular motility, and endergonic redox reactions (Jormakka et al., 2003).

While microorganisms employ a wide range of electron donors and acceptors, their membrane-bound respiratory protein complexes are modular assemblies constructed from a versatile “redox protein construction kit” (Baymann et al., 2003). This toolkit encompasses a limited number of protein units, including catalytic, electron transfer, and membrane anchor proteins (Baymann et al., 2003; Rothery et al., 2008; Simon et al., 2008; Schoepp-Cothenet et al., 2013). Catalytic units facilitate the oxidation or reduction of substrates and typically house transition metal centers with nickel, iron, molybdenum, tungsten, cobalt, and/or copper. In contrast, electron transfer units form an electron-conducting “wire” that shuttles electrons from entry to exit points within a protein complex mainly via multiple [Fe-S] clusters or heme groups. A common representative of such electron transfer proteins is the “four cluster protein” (FCP), containing four cubane [Fe-S] centers, among others found in archetypal complex iron-sulfur molybdoenzyme (CISM) complexes (Rothery et al., 2008). Finally, there are membrane anchor proteins (MAPs) of different types, which primarily anchor the respiratory proteins to the membrane. Additionally, they also participate in the transport of electrons and/or protons across the membrane and thus directly contribute to *pmf* generation (Rothery et al., 2008; Simon et al., 2008; Schoepp-Cothenet et al., 2013).

**Figure 1:**
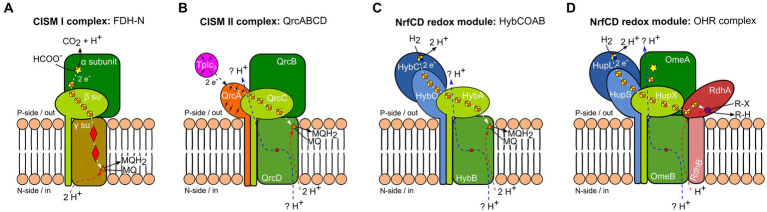
Schematic representation of different anaerobic respiratory protein complexes containing subunits of the complex iron–sulfur molybdenum (CISM) family (Rothery et al., 2008). Homologous proteins and identical cofactors are colored and shaped similarly. White and black arrows indicate electron fluxes, blue and red arrows represent proton fluxes. Electron flux at the p-side of the membrane is accompanied by a proton flux (blue arrow) through two half-channels, linked by a conserved arginine residue (red dot) (Calisto and Pereira, 2021; Duarte et al., 2021). **(A)** Formate dehydrogenase-N (FDH-N) complex of *E. coli* as an example for a type I CISM. Displayed is a monomer from the *in vivo*-associated heterodimeric structure. The large CISM subunit α (dark green), binding *bis*-MGD (yellow star), oxidizes formate at the catalytic site and transfers electrons to heme *b* (red diamonds) of the type I CISM membrane anchor protein (MAP) (γ su, ocher) via the four [4Fe-4S] clusters protein (FCP) (β su, light green) (white arrow). Menaquinone (MQ) is reduced to menaquinol (MQH_2_) at the n-side of the membrane (orange triangle) and takes up two cytoplasmic protons (red arrow) (Jormakka et al., 2002). **(B)** The calculated model of QrcABCD complex of *D. vulgaris* (Duarte et al., 2021) serves as an example of a type II CISM. The large CISM subunit (QrcB, dark green) lacks *bis*-MGD and an [Fe-S] cluster. While there is no experimental proof for proton pumping (blue arrow) through two conserved half-channels (Calisto and Pereira, 2021; Duarte et al., 2021), it has been experimentally shown that electron transfer occurs from type I cytochrome *c*_3_ (TpI*c*_3_, purple) to the QrcA subunit – a membrane-bound cytochrome *c* (orange), FCP (QrcC, light green), reaching the type II CISM MAP (QrcD, intermediate green). This electron transfer leads to the reduction of MQ to MQH_2_ at the p-side of the membrane (orange triangle), accompanied by the uptake of two cytoplasmic protons (red arrow) (Duarte et al., 2018). **(C)** Hydrogenase-2 complex HybCOAB of *E. coli*, with an FCP (HybA, light green) and a proton-translocating type II CISM MAP (HybB, intermediate green). Displayed is homology model of a monomer from the *in vivo*-associated heterodimeric structure (Lubek et al., 2019). The large subunit of the [NiFe] hydrogenase HybC (dark blue), oxidizes hydrogen (H_2_) at the p-side of the membrane. Electrons pass through several [4Fe-4S] clusters of HybO (light blue) and HybA (light green) to the MQ binding-site (orange triangle) of HybB (light green). As has been experimentally shown, MQ protonation is linked to proton transfer from cytoplasm (red arrow) via conserved amino acid residues (Lubek et al., 2019). **(D)** Model of the organohalide respiratory (OHR) complex of *D. mccartyi* belonging to type II CISM complexes. The OHR complex includes the hydrogenase subunits HupL and HupS (dark and light blue), an FCP (HupX, light green), a type II CISM MAP (OmeB, intermediate green), and the large CISM subunit OmeA (dark green) with a putative *bis*-MGD binding site (yellow star), as well as the reductive dehalogenase subunits RdhAB (dark and light red). The [NiFe] hydrogenase HupL oxidizes H_2_ at the p-side of the membrane. Electrons are transferred through several [4Fe-4S] clusters of HupS, HupX, and RdhA subunits to the cobalamin (violet pentagon) bound to the active site of RdhA, where reductive dehalogenation of organohalides occurs.

Respiratory protein complexes exhibit recurring subunit compositions. One example for such a recurrent composition are archetypal CISM complexes, which are composed of three subunits: (i) a large subunit, frequently the catalytic subunit, binding a *bis*-molybdo-pyranopterin guanine dinucleotide (*bis*-MGD) cofactor and a cubane [Fe-S] cluster; (ii) an FCP, basically representing a tandem ferredoxin fold and belonging to the NrfC protein family (Duarte et al., 2021); and (iii) a MAP, belonging either to cytochromes *b* and binding two heme *b* (Figure 1A, CISM type I), or to the NrfD protein family, lacking cofactors (Figure 1B, CISM type II) (Rothery et al., 2008). Another recurring subunit composition in respiratory protein complexes is the co-occurrence of only two of the three CISM-complex subunits: the NrfC-type FCP and the NrfD-type membrane protein, forming a two-membered redox module. This arrangement is observed in type II CISM complexes, often forming multi-subunit membrane complexes, such as the QrcA-D complex (Figure 1B), involved in the hydrogen/formate metabolism of sulfate-reducing bacteria, the ActA-F alternative complex III, involved in oxygen respiration, and the organohalide respiratory (OHR) complex of *Dehalococcoides mccartyi* strains. However, the tight interaction between the NrfC-type FCP and the NrfD-type MAP is not limited to archetypal CISM II complexes but is also found, for instance in *E. coli* hydrogenase-2, where these two subunits are electrically connected to the large and small subunits of a [Ni-Fe] hydrogenase (Figure 1C) (Pinske et al., 2015). Moreover, the NrfCD redox modules can interact with soluble cytochromes *c* without forming a stable protein complex, as seen in NrfA-D nitrite reductase system (Duarte et al., 2021).

Various respiratory chain architectures have evolved, leading to diverse mechanisms for generating the *pmf*. The respiratory chain architecture strongly depends on factors such as the amount of Gibbs free energy change (Δ*G*) achievable from the redox substrate pairs and the difference in standard electrochemical potential (Δ*E*_m_) between the donor and acceptor (Simon et al., 2008; Simon and Kern, 2008; Schoepp-Cothenet et al., 2013; Duarte et al., 2018, 2021). In general, two types of respiratory chain architectures are employed as the bioenergetic system: (i) coupled proton and electron transport via liposoluble proton shuttles, such as quinones, is used when a significant amount of energy is available in the electrochemical potential difference of a substrate pair; and (ii) systems lacking liposoluble carriers, comprised of standalone protein complexes, are mainly used by microorganisms living at the thermodynamic limit (Schoepp-Cothenet et al., 2013).

In quinone-dependent systems, quinones facilitate the transfer of electrons from a donor-oxidizing enzyme to an acceptor-reducing enzyme. Quinones can be reduced and protonated at the cytoplasmic/negative side (n-side) of the membrane, as demonstrated by the example of formate dehydrogenase-N (FDH-N) in *E. coli* (Figure 1A), and move to the positive side (p-side) of the membrane after protonation, where they are re-oxidized by an oxidizing protein complex, releasing protons to the p-side of the membrane. This process of electrogenic protonation and deprotonation of quinones at opposing membrane sites represents a redox-loop mechanism (Mitchell, 1975). In contrast to a redox-loop, protonation and deprotonation of quinones can occur on the same side of the membrane, e.g., at the p-side. This applies to the dissimilatory sulfate reduction pathway, where quinone reduction by the QrcA–D complex (Figure 1B) and quinone oxidation by the DsrMKJOP complex both occur on the p-side of the membrane (Duarte et al., 2021). The key difference is that in FDH-N, electrons are transported across the membrane via cytochrome *b*-type MAP, while in the QrcA-D complex, electrons remain at the p-side, and protons are translocated through the NrfD-type MAP to the p-side via a Grotthuss-type mechanism (Agmon, 1995).

Quinone-independent proton or sodium translocation is a feature observed mainly in microorganisms adapted to low-energy conditions for chemiosmosis. For instance, acetogens employ the RnfA-EG complex, coupling the intracellular oxidation of reduced ferredoxins and subsequent reduction of NAD^+^ to the translocation of protons or sodium ions across the membrane (Kuhns et al., 2020). In methanogens, the methyltransferase MtrA-H complex is responsible for coupling the methyl group transfer from methyl-tetrahydromethanopterin to coenzyme M with sodium ion translocation across the membrane (Gottschalk and Thauer, 2001). Additionally, energy-converting [Ni-Fe] hydrogenases (Ech) achieve quinone-independent proton pumping across the membrane utilizing reduced ferredoxin as electron donor to reduce protons to hydrogen (Katsyv and Müller, 2022). Another notable example of a standalone, quinone-independent respiratory protein complex is the OHR complex in *D. mccartyi* strains (Figure 1D).

*Dehalococcoides mccartyi* strains are strictly anaerobic bacteria, relying on hydrogen as the sole electron donor and halogenated organic compounds as terminal electron acceptor. Most *Dehalococcoides* strains lack genes for quinone (Kube et al., 2005; Seshadri et al., 2005; Schipp et al., 2013), heme, and cytochrome biosynthesis (Löffler et al., 2013; Schipp et al., 2013). Experimental evidence, including growth without quinones, lack of respiration inhibition by HQNO, and failure to detect quinones in the membrane, supports quinone-independent respiration in *D. mccartyi* strains (Jayachandran et al., 2004; Schipp et al., 2013; Kublik et al., 2016). The absence of quinones and cytochromes, coupled with strong OHR subunit expression and subunit interactions identified with blue-native gel electrophoresis indicates that *D. mccartyi* strain CBDB1 relies on a standalone, quinone- and cytochrome-independent respiratory protein complex (Kublik et al., 2016). This respiratory complex is located at the cytoplasmic membrane with membrane-integrated subunits and membrane-associated subunits all facing the periplasmic space of the membrane. *Dehalococcoides* strains have only a single membrane and no peptidoglycan cell wall. Instead they have a proteinaceous surface layer. With the periplasmic space we refer to the space between the membrane and the surface layer. While proteomic data from the membrane fraction of strain CBDB1 identified OHR complex proteins and ATPase subunits in high abundance (Seidel et al., 2018), none of the eleven encoded subunits of a complex I were detected (Kube et al., 2005), indicating that the *pmf* is established independently from complex I (Seidel et al., 2018). The OHR complex of strain CBDB1 is composed of three modules with all active sites oriented towards the p-side of the membrane (Kublik et al., 2016; Seidel et al., 2018). The hydrogen-oxidizing module comprises HupL and HupS, two subunits of a [NiFe] hydrogenase. The central module contains three subunits, similar to an archetypal CISM II complex with OmeA, HupX, and OmeB representing the large subunit, the electron transfer subunit FCP and the MAP, respectively (Kublik et al., 2016; Seidel et al., 2018). The reductive dehalogenase RdhA, together with anchor protein RdhB, forms the output module of the OHR complex, responsible for reductively dehalogenating organic halogenated compounds. The genome of strain CBDB1 encodes 32 different RdhA and RdhB proteins in tightly regulated operons, with the OHR complex incorporating specific reductive dehalogenases based on available electron acceptors. In this study, the cells were grown with 3,5-dibromo-*L*-tyrosin (DBT) or 1,2,4,5-tetrabromobenzene (TeBB) as electron acceptor, which induces the reductive dehalogenases CbrA (locus cbdbA84) and CbdbA80 (Adrian et al., 2007; Wagner et al., 2012) or CbdbA238 and CbdbA1092 (Reino et al., 2023), respectively.

The absence of quinones and cytochrome *b*-type MAP suggests that in *Dehalococcoides* the *pmf* is not established using electron flow through the membrane as in classical redox loops. Instead, the architecture of the OHR complex, featuring the NrfD-type MAP represented by the OmeB subunit, suggests *pmf* generation through direct proton translocation across the membrane. However, the exact mechanism – whether proton pumping (Duarte et al., 2021), vectorial proton transport onto the halogenated substrate (Pinske, 2019), or a combination thereof (Figure 1D) – remains unclear.

The objective of the present study was to investigate the proton translocation mechanism in the OHR complex of strain CBDB1. For that, we conducted labelling experiments with deuterated water to trace the proton paths and matched the results with the outcome of structural modelling. Our findings suggest that the OHR complex achieves energy conservation by conducting electrons through its periplasmic membrane-associated subunits to the halogenated electron acceptor and electrogenic protonation of the reduced electron acceptors with cytoplasmic protons, resulting in the following overall reaction scheme:


$$\begin{array}{l} H_{2}\;\left(\mathrm{outside}\right)+1\;H^{+}\;\left(\mathrm{inside}\right)+R—X\;\left(\mathrm{outside}\right)\to \\ 2\;H^{+}\;\left(\mathrm{outside}\right)+R—H\;\left(\mathrm{outside}\right)+X^{-}\;\left(\mathrm{outside}\right)\text{.} \end{array}$$

In-depth analysis of an *in-silico*-predicted structure for the OHR complex led to the localization of two putative proton-conducting half-channels within OmeB and putative water-filled tunnels that may contribute to proton translocation.

## 2 Materials and methods

### 2.1 Cultivation, cell harvesting, and cell disruption

*Dehalococcoides mccartyi* strain CBDB1 was cultivated in a synthetic liquid medium as previously described (Adrian et al., 2000; Jayachandran et al., 2003). The medium was prepared with either “light” (H_2_O, exclusively) or “heavy” (D_2_O, 80% D_2_O content) water and contained vitamins, trace elements, and 2 mM *L*-cysteine as a reductant. Acetate in a concentration of 5 mM was used as the carbon source, and either 1 mM 3,5-dibromo-*L*-tyrosin (DBT) or 0.5 mM 1,2,4,5-tetrabromobenzene (TeBB) was added as terminal electron acceptor. Cultures prepared with “heavy” water will be referred to as D_2_O cultures, and cultures prepared with “light” water will be referred to as H_2_O cultures. The cultures were incubated at 30°C under strictly anoxic conditions, with the headspace flushed with nitrogen and 0.2 bar hydrogen added as an electron donor. Cells were harvested by centrifugation under anoxic conditions at 9,000 rpm and 16°C for 1 h in 50 mL falcon tubes. Approximately 5% of the initial culture volume was retained as the “cell pellet” for experiments with whole cells. For stronger enrichment, “cell pellets” were combined and centrifuged again under the same conditions. For experiments with crude extracts, 1% (w/v) DDM was added to the harvested cells, which were mechanically disrupted via bead beating at 4 m s^−1^ for 30 s, two times, using a Savant FastPrep-24 homogenizer (MP Biomedicals). Crude extracts were separated from whole cells by centrifugation at 9,000 rpm, 16°C for 10 min under anoxic conditions. The resulting supernatant was used for crude extract experiments.

### 2.2 Experimental design

To investigate the origin of the proton that replaces the halogen substituent of the electron acceptor during reductive dehalogenation, various experimental conditions were tested using methyl viologen-based activity assays. In condition 1, dehalogenase activity assays were conducted with concentrated H_2_O cultures and activity assay buffer prepared with D_2_O. In this condition, cells were saturated with protons inside and deuterium outside (Supplementary Figure S1A). Condition 2 represented the opposite scenario: activity assays were performed with concentrated D_2_O cultures and the activity assay buffer was prepared with H_2_O. Here, cells were saturated with deuterium inside and protons outside (Supplementary Figure S1B). In conditions 3 and 4 the dehalogenase activity assays were conducted using crude extracts obtained from an H_2_O culture. The activity assay buffer in condition 3 contained 20% D_2_O, while in condition 4, it contained 80% D_2_O. Deuterium incorporation into the product via GC-MS was monitored after defined incubation times to distinguish whether the proton or deuterium originated from inside or outside of the cell.

### 2.3 Methyl viologen-based dehalogenase activity assay

To assess dehalogenation activity, a methyl viologen-based activity assay was used under strictly anoxic conditions, as previously described (Hölscher et al., 2003). Methyl viologen was chosen as electron donor due to its significantly higher activity compared to the use of hydrogen (Jayachandran et al., 2004). Triplicate activity assays were prepared in 1.5 mL screw vials. For conditions 1, 3, and 4, 200 μL of cell suspension or crude extract were mixed with 800 μL of an activity assay buffer, resulting in an overall D_2_O content of 20% for condition 3 and 80% for conditions 1 and 4, respectively. For condition 2, 625 μL of intact CBDB1 cells, saturated with 80% D_2_O in the cytoplasm due to the D_2_O content in cultivation medium, were mixed with 375 μL of an activity assay buffer prepared with H_2_O, resulting in a calculated overall D_2_O content of 50% in the vials. The final concentrations of the assay buffer components were 100 mM potassium acetate buffer (pH 5.8), 1 mM methyl viologen as an artificial electron donor reduced with 1 mM titanium(III)citrate, and either 200 μM 2,4,6-tribromophenol (TBP) as a substrate for cells cultured on DBT or 1,2,3,4-tetrachlorobenzene (TeCB) for cells cultured on TeBB. Thus, it contained all necessary components for the transfer of electrons from methyl viologen to the halogenated electron acceptor with the exception of catalyst. The activity test was started by mixing the assay buffer with cell suspension or crude extracts. No-substrate-controls (NSC) and no-enzyme-controls (NEC) were prepared, containing buffer instead of substrate solution in the assay buffer and buffer instead of cells or crude extract suspension, respectively. The 1.5 mL screw vials were sealed and incubated upside down in an anaerobic chamber. After a defined incubation time, reactions were stopped by addition of 10 μL concentrated formic acid, and dehalogenation products were analyzed by gas chromatography, which was coupled to a mass spectrometer (GC-MS).

### 2.4 Sample extraction, derivatization, and GC-MS analysis

The methyl viologen-based dehalogenase activity assay produced 2,4- and 2,6-dibromophenol (DBP) from 2,4,6-tribromophenol (TBP) and 1,2,4-trichlorobenzene (TCB) from 1,2,3,4-tetrachlorobenzene (TeCB). TCB was extracted directly via liquid–liquid extraction with *n*-hexane by adding 500 μL of *n*-hexane to the 1 mL reaction mixture. The hydroxyl group of DBP was derivatized by acetylation prior to GC-MS analysis. For simultaneous derivatization and extraction, 66 μL acetic anhydride, 264 μL *n*-hexane and 170 μL of 0.44 M NaHCO_3_ were added (Ballesteros et al., 1990), and the vials were shaken at 600 rpm for 1 h at room temperature. Subsequently, the upper hexane phase was transferred to fresh 1.5 mL screw vials equipped with 500 μL inserts. A sample volume of 5 μL was injected into a GC (Agilent Technologies 7890A) in splitless mode and separated with a non-polar capillary column BPX5 (SGE; length 30 m, inner diameter 0.25 mm, film thickness 0.25 μm). The injector temperature was set to 280°C. Helium was used as a carrier gas at a flow of 1.74 mL min^−1^. The oven temperature was programmed to rise from 100°C to 250°C at a rate of 20°C min^−1^, and then held at 250°C for 8 min. The GC was coupled to a mass spectrometer (Agilent Technologies 5975C) with the electron multiplier voltage set to 1953 V, and the source and quadrupole temperatures set to 230°C and 150°C, respectively. The mass spectrometer was operated in total ion scan mode between 50 and 550 Da with a solvent delay of 5 min.

### 2.5 Analysis of isotope pattern and calculation of deuteration degree

Deuterium or hydrogen incorporation in product species was distinguished by the specific isotopic pattern of the product isotopologues (Supplementary Figure S2). The shift in the molecular ion peak of DBP from *m*/*z* = 250 (A1) to *m*/*z* = 251 (B1) or of TCB from *m*/*z* = 180 (A1) to *m*/*z* = 181 (B1) indicated the incorporation of deuterium instead of hydrogen. To quantify these shifts, the relative deuteration degree for the first peak pair was calculated. Deuteration degree was defined as the relative amount of the area counts of the deuterated product peak divided by the sum of the area counts of the protonated and deuterated product isotopologues. For DBP this was the relative amount of the DBP product peak at *m*/*z* 251 over the sum of the peaks at *m*/*z* 250 and 251. For TCB this was the relative amount of the peak at *m*/*z* 181 over the sum of the peaks at *m*/*z* 180 and 181 (Eq. 1).


(1) $${DD}_{1}=\frac{B1}{A1+B1}$$

### 2.6 Bioinformatics and protein structure prediction

Amino acid protein sequences were obtained from the National Center for Biotechnology Information (NCBI) Database (Sayers et al., 2022). Protein homology was detected with Conserved Domain Architecture Retrieval Tool (CDART) (Geer et al., 2002) of the NCBI (Sayers et al., 2022). Transmembrane helices were identified by DeepTMHMM version 1.0.24 (Hallgren et al., 2022). To identify conserved amino acids, a multiple sequence alignment of NrfD-like proteins from different bacteria was carried using MEGA 11 (Tamura et al., 2021). The evolutionary relationship between different NrfD-like proteins was inferred using the Maximum Likelihood method, and the evolutionary distances were calculated using Poisson correction (Morgan, 1998). To analyze the evolutionary conservation of OmeB and identify critically important sites within the amino acid sequence, the multiple sequence alignment of 79 homologous proteins was analyzed with the ConSurf server (Ashkenazy et al., 2016). Signal peptide sequences were identified using SignalP 6.0 (Teufel et al., 2022) and were removed from sequences for structure predictions using the AlphaFold2 ColabFold platform (Mirdita et al., 2022). Cofactor binding sites were identified using the COFACTOR server (Roy et al., 2012; Zhang et al., 2017). Subsequent structure refinement, and alignment of the OmeB subunit of strain CBDB1 with the QrcD subunit of *Desulfovibrio vulgaris* were done using ChimeraX (Pettersen et al., 2021). The MOLE 2.0 tool was used to identify tunnels and cavities within proteins (Sehnal et al., 2013). The analysis parameters for the two proteins OmeB and RdhA, respectively were as follows: probe radius 3.6 Å, interior threshold 0.73 Å, minimum depth 1 Å for OmeB and 5 Å for RdhA, bottleneck radius 0.53 Å (OmeB) or 0.4 Å (RdhA), origin radius 1.53 Å (OmeB) or 1 Å (RdhA), bottleneck length 0.65 Å (OmeB) or 1.7 Å (RdhA), and cut off ratio 1 Å for OmeB or 0.5 Å for RdhA.

## 3 Results

### 3.1 Specific activity and turnover rate of the reductive dehalogenase

To investigate the origin of protons incorporated into the dehalogenation products, we conducted methyl viologen-based dehalogenase activity assays under different initial conditions. In conditions 1 and 2, a defined number of intact CBDB1 cells were used for activity tests, with H_2_O initially inside and D_2_O outside (condition 1), or D_2_O initially inside and H_2_O outside the cell (condition 2). In conditions 3 and 4, we prepared crude extracts from a defined number of CBDB1 cells and mixed them with an activity assay buffer containing varying D_2_O concentrations, resulting in setups with either 20% or 80% D_2_O content. To differentiate between the origin of protons in the dehalogenation products, it was crucial to (i) quickly stop the reaction after a few seconds, and (ii) ensure rapid dehalogenation, allowing for measuring proton incorporation into the products before H_2_O and D_2_O exchanged across the cell membrane. Therefore, the dehalogenase activity assay was optimized to achieve quick termination of the reaction after only a few seconds by acidification, enabling the measurement of product formation, e.g., after just 10 s of incubation. For instance, 3.8 ± 0.5 μM DBP were detected after 30 s of incubation in condition 1, while in condition 2, approximately 9.0 ± 0.4 μM DBP were released already after 10 s (Supplementary Figure S4).

**Figure 2:**
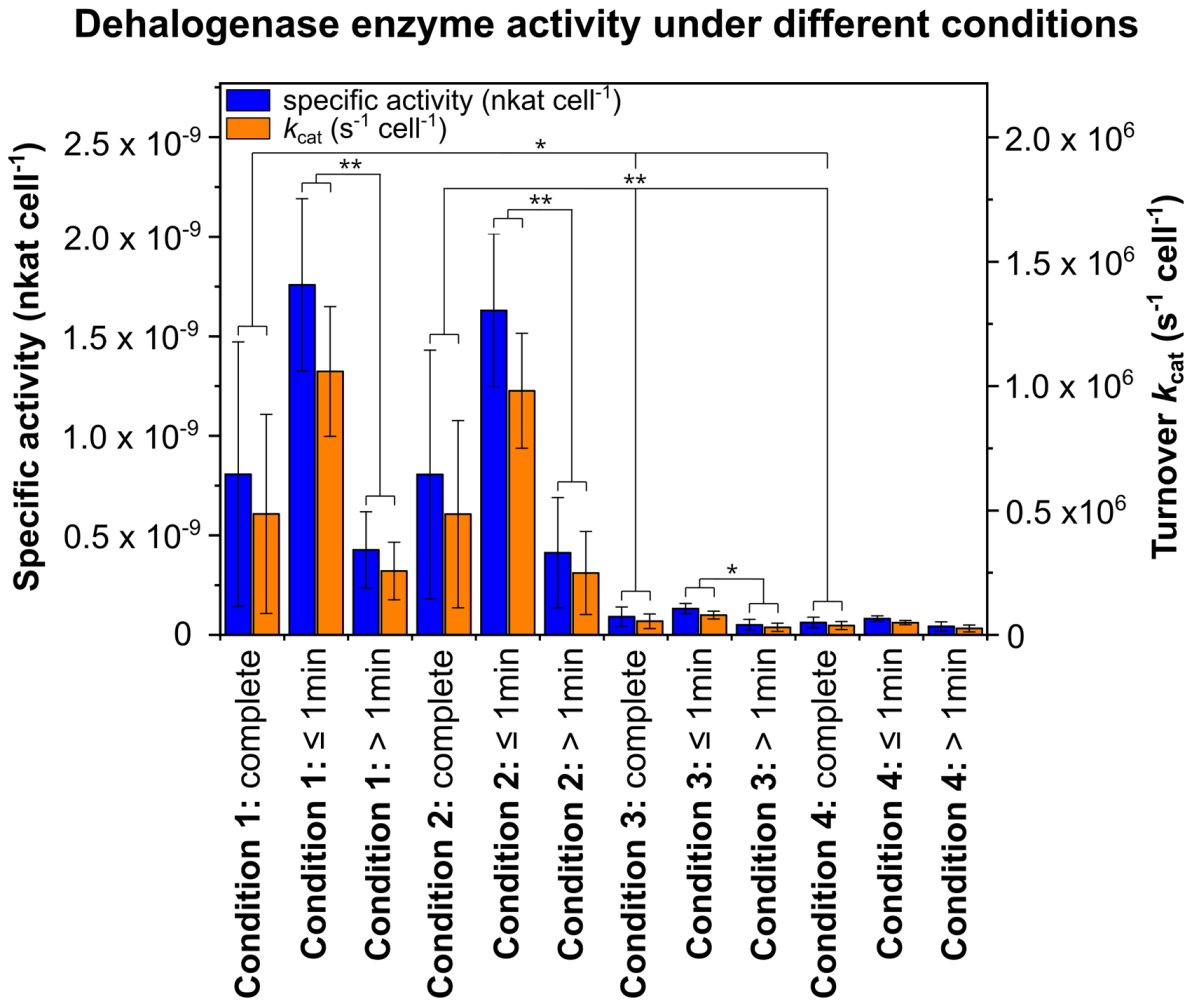
Specific activities per cell (nkat cell^−1^) and turnover rates *k*_cat_ per cell (s^−1^ cell^−1^) of the reductive dehalogenase (RdhA) under different experimental conditions. For condition 1, cells with initially H_2_O inside the cell and D_2_O-based activity assay buffer were used, while for condition 2, cells with initially D_2_O inside the cell and H_2_O-containing activity assay buffer were used. For conditions 3 and 4, crude extracts of CBDB1 cells were used and subjected to activity assay buffer containing 20% or 80% D_2_O. The substrate used, tribromophenol, was present at a concentration of 200 μM and underwent dehalogenation to form dibromophenol (DBP), which was quantified with GC-MS. Activity assays were conducted in triplicates, and the mean values of specific activities and *k*_cat_ per cell as well as standard deviations are shown for the entire incubation period, as well as separately for incubation times ≤1 min or >1 min. Statistical significance was determined using paired two-sample *t*-test and is indicated by stars (*p < 0.05 and **p < 0.01).

Since high specific dehalogenase activity is essential to successfully answer the research question, specific activities per cell (nkat cell^−1^) and turnover rates *k*_cat_ per cell (s^−1^ cell^−1^) were determined for all four conditions. Between conditions 1 and 2 or 3 and 4, no significant differences in specific activities or *k*_cat_ per cell were observed (Figure 2 and Supplementary Table S1). Notably, specific activities per cell and *k*_cat_ cell^−1^ for crude extracts were significantly lower compared to whole cells, averaging about one tenth of the specific activity or *k*_cat_ per cell compared to whole cells. This required the use of higher biomass from a higher overall cell number for setting up activity assays under conditions 3 and 4 to enable detection of product formation after a few seconds. For conditions 1 and 2 as well as for condition 3, specific activities per cell and *k*_cat_ cell^−1^ were 3–4 times higher for incubation times ≤1 min compared to longer incubation times >1 min, and this difference was significant (Figure 2 and Supplementary Table S1).

### 3.2 Deuterium incorporation into dehalogenation products using whole CBDB1 cells

**Figure 3:**
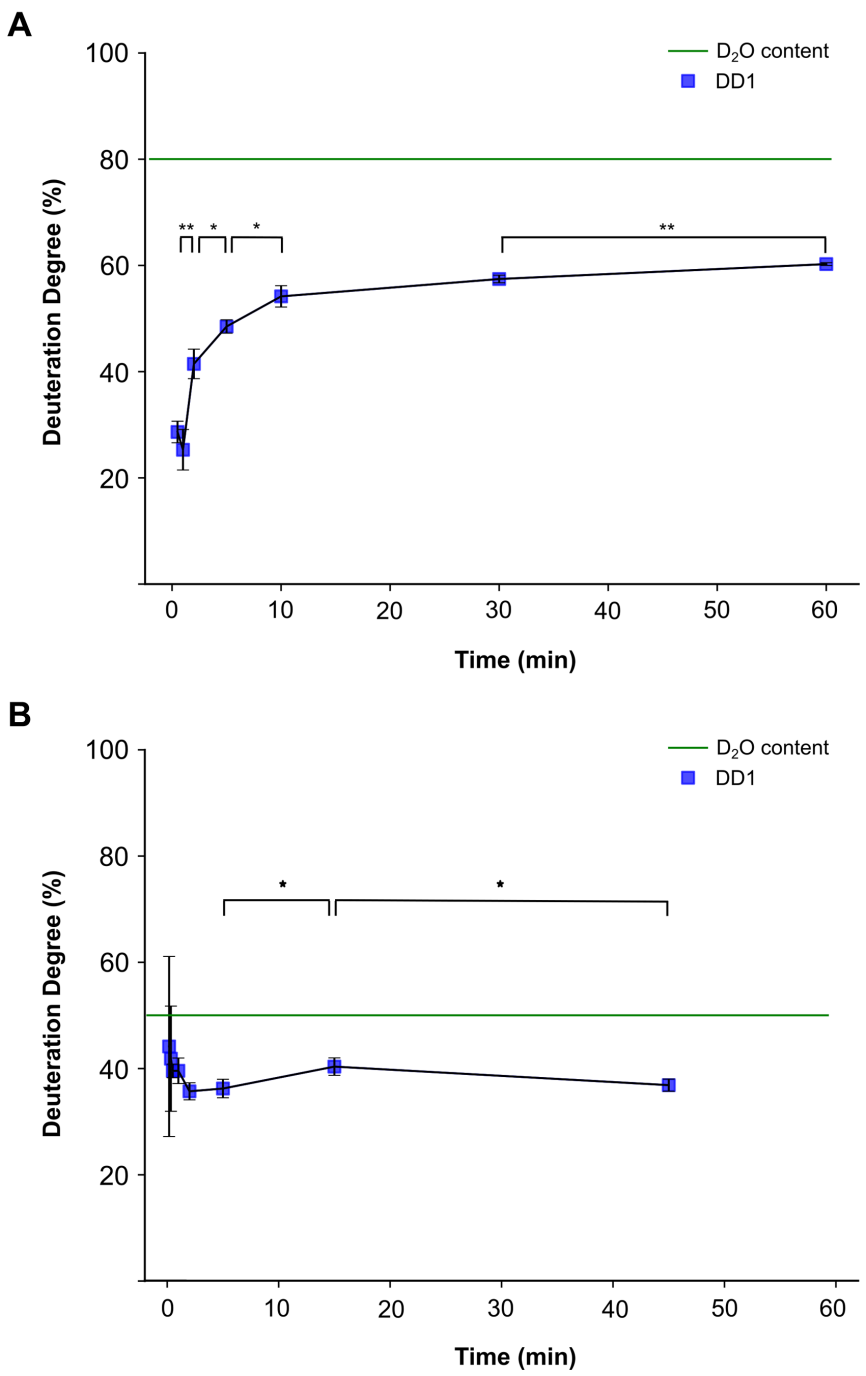
Deuteration degree progression in an *in vitro* activity assay with *Dehalococcoides mccartyi* strain CBDB1 cells cultured with 3,5-dibromo-*L*-tyrosin. The blue squares represent the values of measured deuteration degrees (DD1) of the reaction product dibromophenol after the given incubation times. Tribromophenol was used as the substrate. The activity assays were conducted in triplicates, and the mean values and standard deviations are shown. Statistical significance was determined by paired two-sample *t*-test and is indicated by stars (*p < 0.05 and **p < 0.01). The calculated DD1, which would be reached if cells and activity assay solution were in equilibrium from the beginning of the experiment is represented by the green dashed line. **(A)** DD1 progression for condition 1, in which H_2_O was initially inside the 200 μL of cell suspension and 800 μL of D_2_O-based activity assay buffer was added outside. **(B)** DD1 progression for condition 2, in which D_2_O was initially inside the cell and H_2_O-containing activity assay buffer was used.

To investigate the origin of protons incorporated into dehalogenation products, dehalogenase activity assays were conducted with intact CBDB1 cells under conditions 1 and 2. For condition 1, 200 μL of cell suspension containing approximately 2.9 × 10^8^ cells mL^−1^ were used, while 625 μL of cell suspension containing approximately 6.7 × 10^8^ cells mL^−1^ were used for condition 2. Exemplary results obtained from cells cultured with DBT and subjected to activity assays using TBP as a substrate are described here in detail (Figure 3). The experiment was replicated multiple times with all controls included. Similar behavior was observed in results obtained with cells grown with TeBB as an electron acceptor (Supplementary Figure S3).

For condition 1 with D_2_O initially outside the cell, the deuteration degree started at around 25% within the first minute of incubation and then increased logarithmically (Figure 3A). All data points were consistently lower than the deuteration degree of 80% in the extracellular assay solution, reaching a maximum deuteration degree of approximately 60%. No significant difference in the deuteration degree was observed between the time points of 30 s and 1 min. In contrast, the deuteration degrees for all subsequent time points, except for 10 min and 30 min, were statistically significantly higher than the previous data point with a value of *p* <0.05 (Figure 3A).

Condition 2 was designed as the opposite experiment to condition 1. In this experiment, intact CBDB1 cells containing 80% D_2_O were used along with an activity assay buffer prepared with H_2_O, resulting in a calculated overall value of 50% D_2_O content in the vials. To detect the initial deuteration degree, two early time points of 10 and 20 s were included in the experiments for condition 2. At an incubation time of 10 s, the deuteration degree in the product was approximately 45%. Subsequently, the deuteration degree continuously decreased to a plateau at around 35%, which was reached after approximately 5 min (Figure 3B). At the initial time points of 10 and 20 s, large standard deviations and non-significant differences of deuteration degree shifts between the time points were observed. However, the changes within the first minutes were not significant. The first significant difference with *p* < 0.05 was observed between the incubation times of 5 and 15 min, as well as 45 min. The overall deuteration degree of 50% was not reached at any time.

### 3.3 Deuterium incorporation into dehalogenation products using crude extracts

As a comparison, we conducted further experiments using crude extracts instead of intact cells, where no separation of H_2_O and D_2_O could occur, and therefore no preferential incorporation of protons or deuterium was expected. Crude extracts from H_2_O cultures were used to set up activity assays with activity assay buffer containing either 20% (in condition 3) or 80% D_2_O (in condition 4). For each condition, 200 μL of crude extracts obtained from approximately 3.9 × 10^9^ cells mL^−1^ were used.

**Figure 4:**
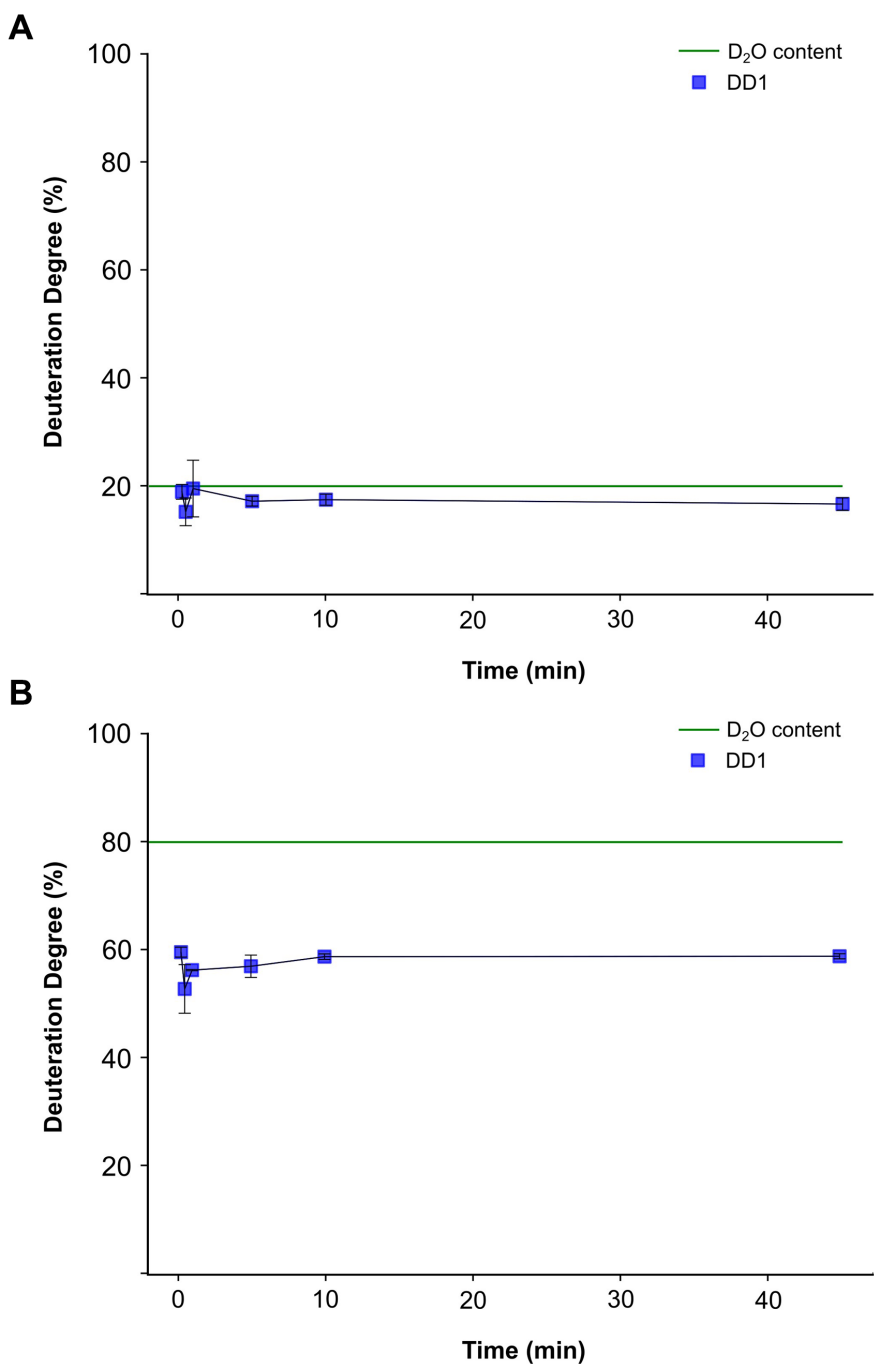
Time course of deuterium incorporation into the dehalogenation product dibromophenol from *in vitro* activity assays conducted with crude extracts obtained from *Dehalococcoides mccartyi* strain CBDB1 cells cultured on 3,5-dibromo-*L*-tyrosin. The blue line represents the detected deuteration degree (DD1) of dibromophenol (DBP) over time. The activity assays were conducted in triplicates, and the mean values and standard deviations are shown. Statistical significance was determined by paired two-sample *t*-test. No significant difference could be determined between the individual measurement time points. The dashed green line indicates the theoretical DD1 at equilibrium. **(A)** DD1 progression of DBP for condition 3, where D_2_O equilibrium of 20% is expected. **(B)** DD1 progression of DBP for condition 4, where D_2_O equilibrium of 80% is expected.

For both conditions, the deuteration degree in the product remained nearly constant over the entire incubation period (Figure 4). In condition 3, the deuteration degree in the product ranged between 16 and 18% (Figure 4A), nearly reaching the overall assay deuterium content of 20% at equilibrium. However, in condition 4, a deuteration degree between 55 and 58% was observed (Figure 4B), significantly lower than the applied deuterium content of 80% in the assay. Notably, the discrepancy between the determined product deuteration degree and the assay deuterium content was much larger for condition 4 than for condition 3. No significant differences in deuteration degrees were observed between the individual time points under condition 3 and condition 4.

In conclusion, our experiments demonstrated that the deuteration degree progression of the product was not time-dependent when crude extracts were used, unlike experiments with whole cells (Figure 3). However, a consistent preference for protons over deuterium ions in the incorporation of dehalogenation products was observed. This preference is evident as the theoretical deuterium content of 80% used in the activity assay buffer of conditions 4 was never achieved (Figure 4B), although no significant differences in total product formation (Supplementary Figure S4B) and no significant differences in specific activities or *k*_cat_ per cell between conditions 3 and 4 were observed (Figure 2 and Supplementary Table S1).

### 3.4 Amino acids in the OmeB subunit of the OHR complex potentially involved in proton transfer

To assess the potential of the OmeB subunit in the OHR complex for proton transport across the membrane, we conducted sequence homology analysis, multiple sequence alignment, and structure prediction. The analysis using the DeepTMHMM online platform predicted ten transmembrane helices (TMHs) in the OmeB subunit from strain CBDB1 (Supplementary Figure S5A), with no N-terminal signal peptide, as had been predicted by SignalP 6.0. Thus, OmeB is an integral membrane protein with a molecular weight of approximately 44.8 kDa consisting of 403 amino acids. The CDART server analysis revealed strong sequence similarity between OmeB and proteins of the NrfD family, known for their role in translocating protons across the membrane, and thus contributing to energy conservation by transmembrane charge separation through quinone reduction at the p-side and electrogenic proton uptake from the cytoplasm (Venceslau et al., 2010; Duarte et al., 2018; Lubek et al., 2019).

The multiple sequence alignment of 23 OmeB homologous proteins identified several conserved amino acid residues, including glutamate, aspartate, histidine, tryptophan, lysine, arginine, serine and threonine. All these residues are capable of breaking and forming hydrogen bonds and the majority of them are located within the TMHs, giving them the potential to be involved in proton translocation across the membrane. However, a handful of conserved amino acids is situated at the entry or exit regions, directing towards the cell interior or exterior, respectively. While not all identified amino acids are highly conserved among all OmeB homologs, functional conservation can be inferred at several positions (Supplementary Figure S5A). Notably, a highly conserved arginine residue (replaced by lysine in *Desulfovibrio vulgaris* and *Candidatus* Avidesulfovibrio excrementgallinarum), proposed to act as a gatekeeper (Calisto and Pereira, 2021; Duarte et al., 2021) was found at the C-terminus in 19 out of 23 OmeB homologs (Supplementary Figure S5A). Phylogenetic analysis revealed that OmeB of *Dehalococcoidia* clusters with the HybB subunits of *Dehalogenimonas* sp. WBC-2 and *E. coli* as well as with the QrcD subunits of *Desulfovibrio vulgaris* and *Pseudodesulfovibrio portus* (Supplementary Figure S5B).

**Figure 5:**
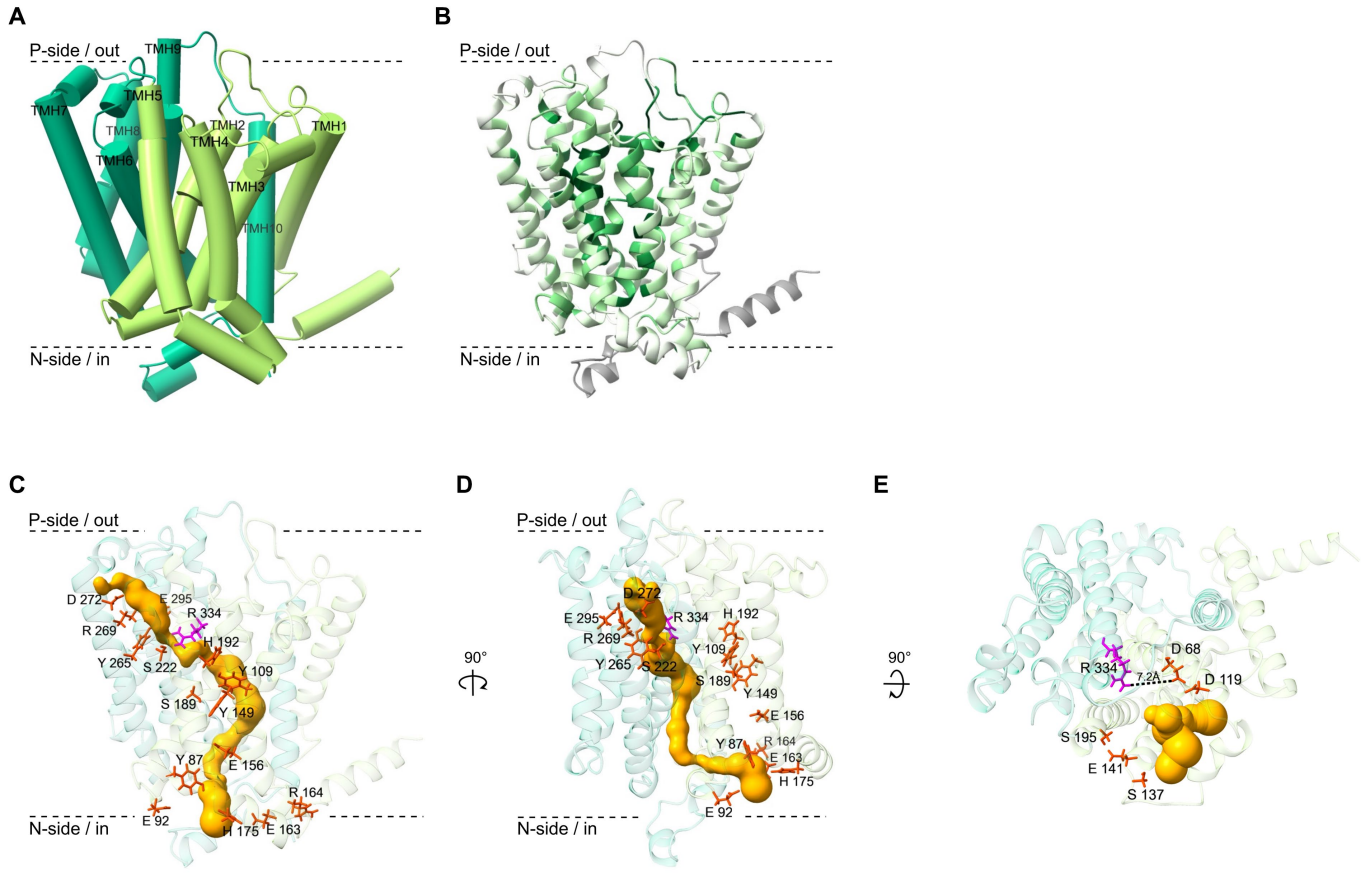
Structure and functional sites of the OmeB protein from *Dehalococcoides mccartyi* strain CBDB1 investigated with AlphaFold2 and other computational methods. **(A)** Ribbon diagram of the OmeB backbone, with transmembrane helices 1–5 depicted in light green and transmembrane helices 6–10 in dark green. The helices are numbered from N- to C-terminus. **(B)** The ConSurf server was used to calculate the conservation level of 79 OmeB homologous amino acid sequences, which is represented by a color scaling scheme ranging from dark green for highly conserved regions to bright green for variable regions. Regions for which the conservation level could not be determined are shown in gray. **(C)** Amino acid residues of OmeB forming the two half-channels putatively involved in proton translocation are shown in stick conformation (red and magenta). The highly conserved Arg334 in the center of the two potential half-channels is highlighted in magenta. A predicted water-filled tunnel spanning OmeB calculated by MOLE is shown in gold. **(D)** The OmeB structure rotated by 90° to the right around the y-axis. Colors as in panel C. **(E)** The OmeB structure rotated by 90° to the front around the *x*-axis. Amino acids in red stick representation show highly conserved amino acid residues constituting the putative quinone binding site (Q-site). The conserved Arg334, oriented towards the Q-site, is shown in magenta. The gold color illustrates the putative water-filled cavity forming the Q-site pocket, calculated using MOLE.

Using AlphaFold2, we calculated the protein structure of OmeB with an overall confidence score of 94.5. The model indicates that the ten TMHs are arranged in two four-helix bundles (TMHs 2–5 and TMHs 6–9), with two additional helices (TMH 1 and TMH 10) at the N- and C-terminus, respectively (Figure 5A). Analyzing evolutionary conservation and identifying critical sites within the proteins using the ConSurf server, we found that the TMHs 2–5 of OmeB homologs contain highly conserved regions (Figure 5B; Supplementary Figure S5C), while TMHs 6–9 exhibit lower overall conservation but carry several conserved amino acids, particularly facing the p-side of the membrane, and TMH 6 contains highly conserved amino acids, especially in the region oriented towards TMHs 2–5 (Figure 5B; Supplementary Figure S5C).

In the AlphaFold2-predicted OmeB protein structure of strain CBDB1, highly conserved amino acid residues identified by the multiple sequence alignment form two half-channels spanning the protein from the n-side to the p-side of the membrane. The n-side/cytoplasmic half-channel is located in the first four-helix bundle of TMHs 2–5 and is formed by Tyr87, Glu 92, Tyr109, Tyr149, Glu156, Glu163, Arg164, His175, Ser189, and His192. The p-side/periplasmic half-channel, consisting of Ser222, Tyr265, Arg269, Asp272, Glu295, and Arg334 is located in TMH 6–9. The two putative half-channels of OmeB converge at the highly conserved Arg334 residue (Figures 5C,D).

OmeB proteins of different *D. mccartyi* strains exhibit high sequence similarity, and amino acids proposed to form the two half-channels are 100% conserved in all seven aligned OmeB proteins (Supplementary Figure S5A). The amino acids implicated in constituting the two half-channels in OmeB also show a high level of conservation of approximately 70% compared to QrcD, a protein with extensively described function and structure (Calisto and Pereira, 2021; Duarte et al., 2021). Therefore, we superimposed the AlphaFold2 OmeB structure with the QrcD homology-based structure (obtained with AlphaFold2) as well as with the experimental structure of the ActC subunit of the alternative complex III (Sousa et al., 2018) (overlays not shown), and found putative proton conducting amino acid residues at the same spatial position as described for QrcD and ActC, respectively despite the absence of a stringent conservation pattern in the half-channel forming amino acid residues of NrfD-like proteins.

To explore the potential role of water-filled pockets or tunnels in facilitating proton translocation by OmeB, we employed the MOLE 2.0 tool. Our analysis revealed several inner cavities also spanning OmeB from the outer to the cytoplasmic side of the membrane, with a maximum length of around 90 Å. The location of the p-side tunnel corresponds to the position of the amino acids that form the p-side half-channel in OmeB (Figure 5C). Interestingly, the second tunnel oriented to the n-side of the membrane runs behind the n-side channel (Figure 5D). Notably, both tunnels and the two half-channels converge at Arg334. At this location, the tunnel has a radius of approximately 0.8 Å, which is smaller than that of a water molecule. The n-side tunnel of OmeB contains two regions with a radius greater than 1.4 Å, sufficient for a water molecule passage, and one bottleneck region with a radius measuring only 0.6 Å. The p-side tunnel has a more bulbous shape, with the largest radius measuring about 2 Å.

### 3.5 Putative quinone binding pocket in the OmeB subunit of the OHR complex

Although all experimental evidence suggests that the OHR complex of *D. mccartyi* strain CBDB1 functions independently from quinones (Jayachandran et al., 2004; Löffler et al., 2013; Schipp et al., 2013; Kublik et al., 2016), the predicted OmeB structure, when superimposed with the quinone-dependent homology-based QrcD and experimental ActC structures, suggested the presence of a quinone binding pocket at the p-side of the membrane (Figure 5E) (Simon and Kern, 2008; Sousa et al., 2018; Calisto and Pereira, 2021). Additionally, MOLE analysis revealed the presence of a bifurcating tunnel originating at the Q-site, forming two branches: (i) a narrow tunnel between TMH 3 and 4 guiding towards the inner space of the membrane, and (ii) a second tunnel extending towards the p-side (Figure 5E). Although the Q-site pocket’s surrounding is mainly composed of hydrophobic and non-conserved amino acids, such as valine, isoleucine, leucine, and methionine, the multiple sequence alignment of OmeB homologs demonstrated high conservation for several protonatable amino acids in the direct vicinity of this binding pocket, including Asp68, Asp119, Ser137, Glu141, and Ser195 (Figure 5E; Supplementary Figure S5A). Asp68 and Asp119 form the back end of the binding site (Supplementary Figure S6B), with Asp68 positioned approximately 7.2 Å away from the highly conserved Arg334.

## 4 Discussion

In this study, we provide evidence supporting the hypothesis that the OHR complex of *D. mccartyi* strain CBDB1 functions as a quinone-independent, protein-based respiration complex, coupling periplasmic electron flux with proton translocation across the cytoplasmic membrane. Our experimental data indicate that, during enzymatic dehalogenation catalyzed by RdhA, protons from the cytoplasm are incorporated into the dehalogenation product in the extracellular space, thereby contributing to a *pmf*. By comparing the calculated structural model of OmeB with the calculated structural model of QrcD and the experimental structure of ActC, we have gathered evidence that OmeB might facilitate proton translocation through a hydrogen-bonded chain mechanism. In this case, RdhA and its halogenated substrate might act as a substitute for a quinone at the p-side of the membrane by accepting two electrons from the periplasmic electron transport chain and one proton from the cytoplasm that is guided to the halogenated electron acceptor. Consequently, our findings reveal an elegantly simple proton translocation mechanism in the OHR complex.

### 4.1 The proton incorporated into the dehalogenation product originates from the cytoplasm

Conditions 1 and 2 were designed to explore the effect of the initial localization of D_2_O inside or outside the cell on the deuteration degree of the dehalogenated product. Our results indicate fast equilibration of water across the membrane (within approximately 10 min), but leaving a sufficient time window to detect differences between different conditions within the initial minutes of incubation. We have not investigated the reason for this relatively slow equilibration but assume that it is due to the unusual membrane composition of *Dehalococcoides* cells that contain mostly saturated fatty acids (White et al., 2005). However, this necessitated a highly active enzyme. The substantial turnover rate of about 1 million molecules per cell and second (Figure 2 and Supplementary Table S1) allowed such fast measurements. With an estimated count of around 100 RdhA proteins per cell (Schiffmann et al., 2014), this translates to an approximate turnover of ~10,000 molecules per second per protein.

Our results suggest that the proton incorporated into the dehalogenated product originates from the cytoplasm. Specifically, under condition 1, where D_2_O was outside and H_2_O was inside the cell, the dehalogenase activity assay showed that mainly protons and not deuterium ions were incorporated into the dehalogenated product at the onset of the reaction (Figure 3A; Supplementary Figure S3A). As the incubation time progressed and D_2_O diffused into the cytoplasm more deuterium was incorporated into the reaction product. In contrast, experiments conducted with crude extracts obtained from cells grown on H_2_O-containing medium and an activity assay buffer composed of either 20% D_2_O or 80% D_2_O (condition 3 or 4) showed a linear course of deuteration degree (Figure 4), highlighting the importance of the intact cell membrane and its respiratory protein complexes for the observations in Figure 3A and Supplementary Figure S3A.

Under condition 2, where H_2_O was outside and D_2_O was inside the cell, there was no preferential initial incorporation of protons or deuterium ions, as observed in condition 1. Instead, we only observed non-significant changes in the deuteration degree over the entire incubation period (Figure 3B; Supplementary Figure S3B). This seems to contradict the explanation that protons/deuterium ions were taken up from the cytoplasm but this effect can be explained by a significantly lower conductivity for deuterium through membrane proteins, a common phenomenon in proton-translocating membrane proteins due to high kinetic isotope effects caused by deuterium, having twice the mass of protons (Hallen and Nilsson, 1992; Karpefors et al., 1999; Salomonsson et al., 2008). A detailed discussion of this effect can be found in the Supplementary Section 3.1, entitled “The OHR complex discriminates against deuterium translocation across the membrane”. The absence of a low initial deuteration degree at the onset of the reaction and the absence of a subsequent increase in condition 2, indicates that protons are not incorporated from the outside into the dehalogenation product. It is important to notice that this evidence is not impacted by the deuterium ion fractionation effect. If the protons do not originate from the outside, they must come from the inside of the cell, supporting the results under condition 1 (Figure 3A; Supplementary Figure S3A). In future studies, the proton transport coupled to dehalogenation should be validated using additional methods such as intracellular pH measurements or ATP quantification. However, due to low biomass available such studies are currently out of reach.

We obtained essentially the same results with different cultures that had been grown with different halogenated electron acceptors and that had expressed different RdhA proteins (CbdbA238 and CbdbA1092 in cultures grown on DBT, Figure 3; CbdbA84 and CbdbA80 in cultures grown on TeBB, Supplementary Figure S3). Therefore, our findings demonstrate that the mechanism of proton translocation in the OHR complex is conserved, regardless of the specific dehalogenases present in the complex, suggesting that the OHR complex can adapt to different growth substrates while maintaining its fundamental mechanism of proton translocation.

### 4.2 Proton translocation in the OmeB subunit is mediated by the hydrogen-bonded chain mechanism

Based on the homology of OmeB to NrfD-like proteins, it was proposed that OmeB is responsible for the proton-transducing activity inside the OHR complex (Duarte et al., 2021). In the calculated model of OmeB, we have identified n- and p-side half-channels, which are characteristic for NrfD proteins (Sousa et al., 2018; Calisto and Pereira, 2021; Duarte et al., 2021), along with two putatively water-filled tunnels with a maximal diameter of 2.5 Å (Figures 5C,D). Tunnels with diameters above 1.4 Å could allow for the passage of water molecules, suggesting that the inner cavities of OmeB may contain water participating in proton conduction. Membrane proteins with ordered water molecules, such as in complex I (Baradaran et al., 2013; Parey et al., 2021), are considered to be crucial for proton translocation via the hydrogen-bonded chain mechanism. The crystal structure of PsrC from *Thermus thermophilus*, a member of the NrfD family, has also revealed the presence of ordered water molecules in the central region of the protein (Jormakka et al., 2008).

**Figure 6:**
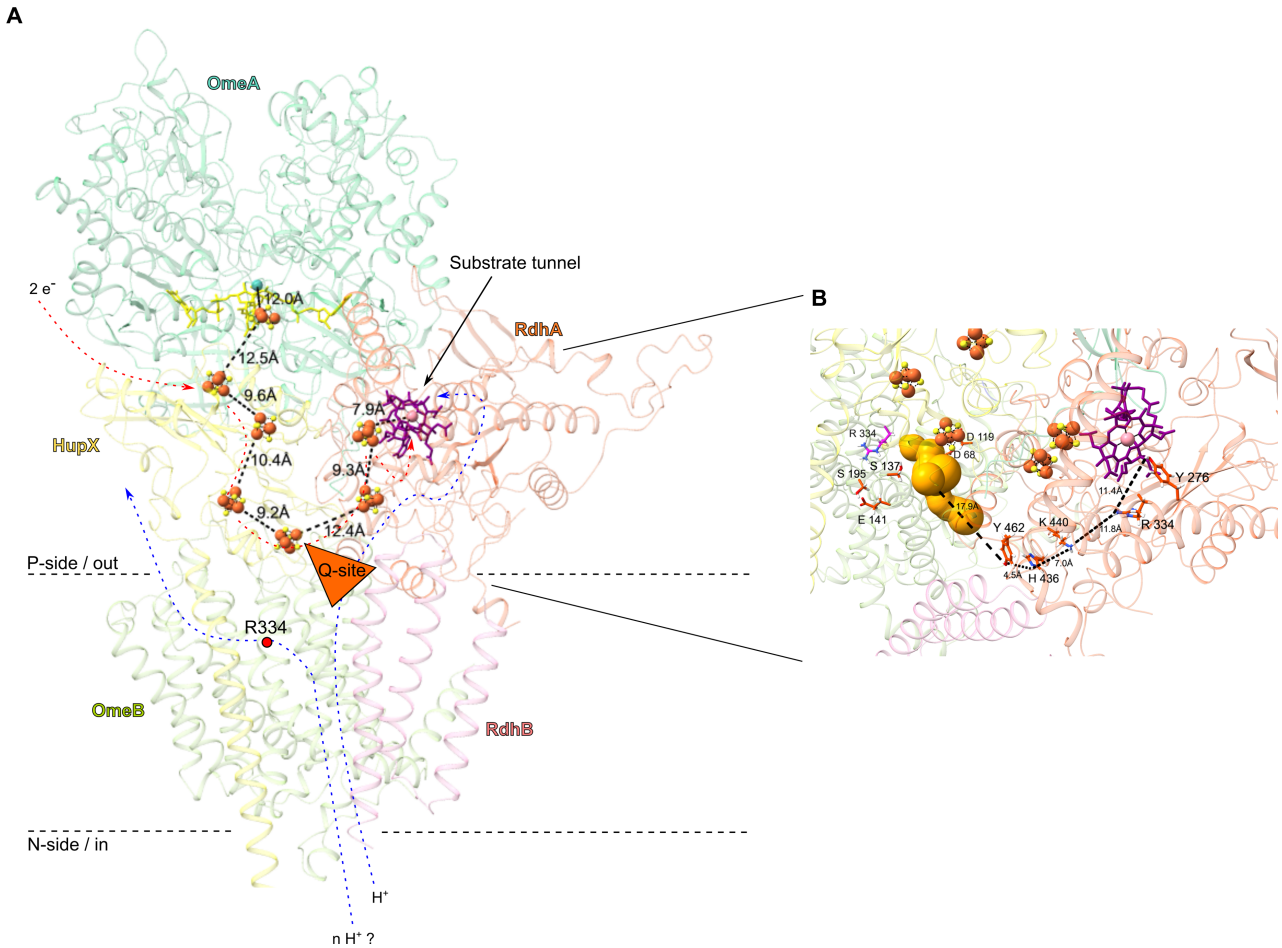
OmeAB-HupX-RdhAB subunits of the OHR complex from *Dehalococcoides mccartyi* strain CBDB1, highlighting conserved amino acid residues and cofactors. **(A)** Overview in cartoon representation. The structures of OmeAB-HupX and RdhAB (encoded by loci cbdbA84 and cbdbA85) were computed using AlphaFold2. Distances in Ångström between metallocofactors are shown. The OmeA subunit (dark green) is predicted to contain a [3Fe-4S] cluster and a *bis*-MGD cofactor (yellow, stick conformation), while the ferredoxin-like HupX subunit (yellow) anchors to the membrane with one transmembrane helix and binds four [4Fe-4S] clusters arranged in an electron-conducting “wire”, with its distal [4Fe-4S] cluster in close proximity of *ca*. 10 Å to the putative Q-site (orange triangle) in the OmeB subunit (light green). The RdhA subunit (orange), anchored to the membrane by RdhB (pink), contains a cobalamin cofactor (purple, stick conformation) and two [4Fe-4S] clusters, and its active site is accessible through a substrate tunnel with a length of approximately 12 Å. Electron flux through the [4Fe-4S] clusters could induce proton translocation across the OmeB subunit (blue arrows), either through the Q-site to the active site of RdhA or to the opposite site of the Q-site. The direction of proton flow is probably controlled by the strictly conserved Arg334 in OmeB (red dot). **(B)** Zooming in on the putative proton translocating region of OmeB and RhdA. Several conserved amino acid residues capable of establishing and breaking hydrogen bonds were identified in this region, forming a continuous proton-conducting path between the Q-site (gold bubbles) and the active site of RdhA. These amino acids are shown in red stick representation, and the distances between them and the Q-site are depicted.

The AlphaFold2-predicted OmeB structure suggests the presence of a putative quinone binding pocket (Q-site), potentially filled with water (Figure 6), although strain CBDB1 lacks quinones and cytochromes (Jayachandran et al., 2004; Schipp et al., 2013; Kublik et al., 2016). However, the prediction of a Q-site in the predicted OmeB structure does not necessitate quinone binding and may be an artifact resulting from the structural models used to train the AlphaFold2 neural network. Nevertheless, we identified several highly conserved protonatable amino acid residues surrounding the putative Q-site of OmeB (Figure 5E), which could play a role in transporting protons from OmeB to RdhA without the involvement of quinones.

Two periplasmic exits for protons may be formed in OmeB: (i) the p-side half-channel and (ii) the putative Q-site. The conserved amino acids of the p-side half-channel align with the putative p-side water-tunnel (Figure 5C), while the n-side half-channel and the n-side water-tunnel run in parallel until they converge at the central gatekeeper amino acid residue Arg334 (Figure 5D). The first four-helix bundle of OmeB, housing the amino acids of the n-side half-channel, exhibits a stronger conservation degree than the four-helix bundle containing the p-side half-channel (Figure 5B; Supplementary Figure S5C), suggesting that the n-side half-channel might be evolutionarily older. Arg334 might play a role in switching the proton exit directing protons to either the p-side half-channel or the Q-site (Calisto and Pereira, 2021). We propose that OmeB may exist in two distinct states, governed by the Arg334 switch:

1. *Q-site-on-state*: in this state, Arg334 connects the n-side half-channel with the putative Q-site. Protons from the cytoplasm might travel along the n-side half-channel to the Arg334 switch. Then they could enter the Q-site with its conserved protonatable amino acids Asp68, Asp119, Ser137, Glu141, and Ser195. From here, proton transfer to RdhA can occur (Figure 6B). The proton exit through the p-side half-channel might be obstructed due to the narrow water-tunnel around Arg334, having a diameter of only 0.8 Å, making it impassable for water molecules. Proton transfer through the p-side half-channel might only occur after a conformational change or rotation of Arg334.
2. *Q-site-off-state*: in this state, Arg334 rotates to connect the p-side half-channel to the n-side water channel, which allows protons to exit through the p-side half-channel. In this state, proton transfer to the Q-site is blocked.

In our experiments, the *Q-site-on-state* in OmeB was likely observed, possibly induced by the reduction of the corrinoid cofactor, either through methyl viologen as previously described (Parthasarathy et al., 2015) or via the [4Fe-4S] cluster. The reduced corrinoid cofactor initiates substrate reduction in the active site of RdhA. Subsequently, the reduced halogenated compound induces proton suction, abstracting a proton from the neighboring amino acid in the active side (Tyr276), leading to subsequent conformational changes that “switch on” the n-side half-channel proton pathway and the outward movement of protons from the cytoplasm via a “hop-turn” mechanism. A similar observation has been made for proton translocation in HybB and QrcD, where quinone binding induces a conformational change (Duarte et al., 2018; Lubek et al., 2019; Parey et al., 2021). However, conditions that induce the *Q-site-off-state* remain unclear, and experimental evidence for this output triggering and the potential for NrfD proteins to act as a “proton pump” have not been provided yet (Jormakka et al., 2008; Sousa et al., 2018; Sun et al., 2018).

### 4.3 RdhA facilitates proton flux to active site through conserved protonatable amino acid residues

To investigate proton conduction between OmeB and the active site of the RdhA, we modelled the overall structure of the OmeAB-HupX-RdhAB complex (Figure 6). For that we individually computed RdhAB (CbdbA84 and CbdbA85) and compared it to the previously published *Dh*PceAB complex of *Desulfitobacterium hafniense* (Supplementary Figure S7) (Cimmino et al., 2023). Our computational analysis indicated convergence between OmeB’s putative Q-site and HupX’s distal [4Fe-4S] cluster, suggesting electrons and protons might concur at this position. RdhA’s interaction with OmeAB-HupX is likely at this juncture. Vital to this mechanism and based on our OHR complex knowledge (Kublik et al., 2016; Seidel et al., 2018; Pinske, 2019), we computationally docked RdhAB to OmeAB-HupX to enable a freely accessible substrate tunnel from the periplasm, with RdhA’s distal [4Fe-4S] cluster situated 12 Å apart from the distal [4Fe-4S] cluster of HupX (Figure 6A). This configuration would facilitate efficient electron transfer between individual metallocofactors in HupX and RdhA (Page et al., 1999). The conserved residues around the putative Q-site might enable proton transfer to the RdhA, which is efficient over distances of around 10 Å (Mikulski and Silverman, 2010; Bondar, 2022). Similar amino acid residues have been implicated in proton transfer in QrcD of *D. vulgaris* (Calisto and Pereira, 2021; Duarte et al., 2021) and HybB in the HybABOC hydrogenase complex of *E. coli* (Lubek et al., 2019).

Based on our analysis of the RdhA homology-based structure and multiple sequence alignment of all 32 reductive dehalogenase amino acid sequences of strain CBDB1 (Supplementary Figure S6), we found conserved protonatable amino acids capable of forming a continuous proton transport path also within the RdhA. Specifically, Tyr276, Arg334, His436, Lys440 and Tyr462 were highly conserved in all 32 RdhAs from strain CBDB1 as well as in PceA from *Sulfurospirillum multivorans* and *Dh*PceA from *D. hafniense* (Supplementary Figure S7B). AlphaFold2 structure analysis suggests that these amino acid residues might drive proton transport within the RdhA, constituting an uninterrupted path from OmeB’s Q-site to RdhA’s active site (Figure 6B), as previously indicated for Tyr276 and Arg334, situated near the corrinoid cofactor in PceA from *S. multivorans* (Bommer et al., 2014) and *Dh*PceA from *D. hafniense* (Cimmino et al., 2023). In addition to the potentially water-filled substrate tunnel in RdhA, which was similarly identified for *Dh*PceA (Cimmino et al., 2023), our MOLE analysis identified several potential water-filled cavities with diameters up to 2.4 Å on the side of RdhA facing OmeB near His436 and Tyr462 (Supplementary Figure S6B), which could facilitate auxiliary proton transfer between OmeB and RdhA through water molecules. Notably, water-filled cavities have been also detected within the reductive dehalogenase PceA of *S. multivorans* (Bommer et al., 2014). Considering the possible link between RdhA’s water-filled tunnel and conserved amino acid residues at OmeB’s Q-site, it is conceivable that electron transport along the [4Fe-4S] cluster wire could initiate proton transfer through OmeB. Moreover, a scenario exists wherein a segment of RdhA or RdhB extends into OmeB’s quinone binding region, as observed in QrcD where a tyrosine residue from QrcC contributes to Q-site formation (Duarte et al., 2021).

In summary, our study indicate that the OHR complex in *D. mccartyi* strain CBDB1 couples exergonic electron flow through periplasmic membrane-associated subunits to the endergonic export of protons against the *pmf*. In contrast to other members of the NrfD protein family, such as QrcABCD of sulfate-reducing bacteria (Venceslau et al., 2010; Duarte et al., 2018) and HybOCAB of *E. coli* (Lubek et al., 2019) achieving energy conservation by electrogenic protonation of quinones at the p-side, the OHR complex might achieve energy conservation by electrogenic protonation of organohalides at the p-side. This coupling of periplasmic electron flow to proton flux across the membrane is a very simple mechanism of *pmf* generation, and might represent a mode of ancestral *pmf* generation. By employing computational tools, we found a potential pathway for proton flux through OmeB to the active site within the reductive dehalogenase subunit. However, we cannot rule out that the OHR complex “pumps” additional protons into the periplasm through other mechanisms.

## Data availability statement

The original contributions presented in the study are included in the article/Supplementary material, further inquiries can be directed to the corresponding author.

## Author contributions

NH: Data curation, Investigation, Methodology, Writing – original draft. ME: Data curation, Investigation, Methodology, Writing – original draft. MP: Data curation, Investigation, Methodology, Writing – original draft. SK: Methodology, Supervision, Validation, Writing – review & editing. OE: Validation, Writing – review & editing. DD: Conceptualization, Supervision, Validation, Writing – review & editing. LA: Conceptualization, Supervision, Validation, Writing – review & editing.
