# Supplementary material for: Dehalococcoides mccartyi strain CBDB1 takes up protons from the cytoplasm to reductively dehalogenate organohalides indicating a new modus of proton motive force generation
Source: Front Microbiol. 2023 Dec 22;14:1305108. doi: 10.3389/fmicb.2023.1305108 (PMC10772276; doi:10.3389/fmicb.2023.1305108)
Supplement: Supplementary file 1 [file Data_Sheet_1.pdf]

Supplementary material to the manuscript:

Dehalococcoides mccartyi performs electrogenic organohalide reduction by taking up protons from the cytoplasm

1 SUPPLEMENTARY MATERIALS AND METHODS

1.1 Experimental design

To investigate the source of protons involved in the substitution of halogen substituents during reductive dehalogenation, controlled experimental assays were conducted. In condition 1, CBDB1 cells saturated with H_2O were subjected to dehalogenase activity assays using an activity master mix prepared with D_2O (Supplementary Figure 1 A). Conversely, in condition 2, CBDB1 cells grown on D_2O-saturated medium were used, and the activity master mix was prepared with H_2O, resulting in internal saturation of cells with deuterium and external exposure to protons (Supplementary Figure 1 B). The incorporation of protons or deuterium into the dehalogenation products was monitored over specific incubation periods during an in vitro dehalogenase enzyme activity assay. A prediction model, as depicted in Supplementary Figure 1, illustrates the expected progression of deuteration degree in the products based on the source of protons. It is worth noting that these observations are limited to a short period of time before the equilibrium of H_2O/D_2O is reached by diffusing through the cytosolic membrane. To ensure accurate results, it was essential to maintain a high specific activity of the reductive dehalogenase and rapidly terminate the enzymatic activity.

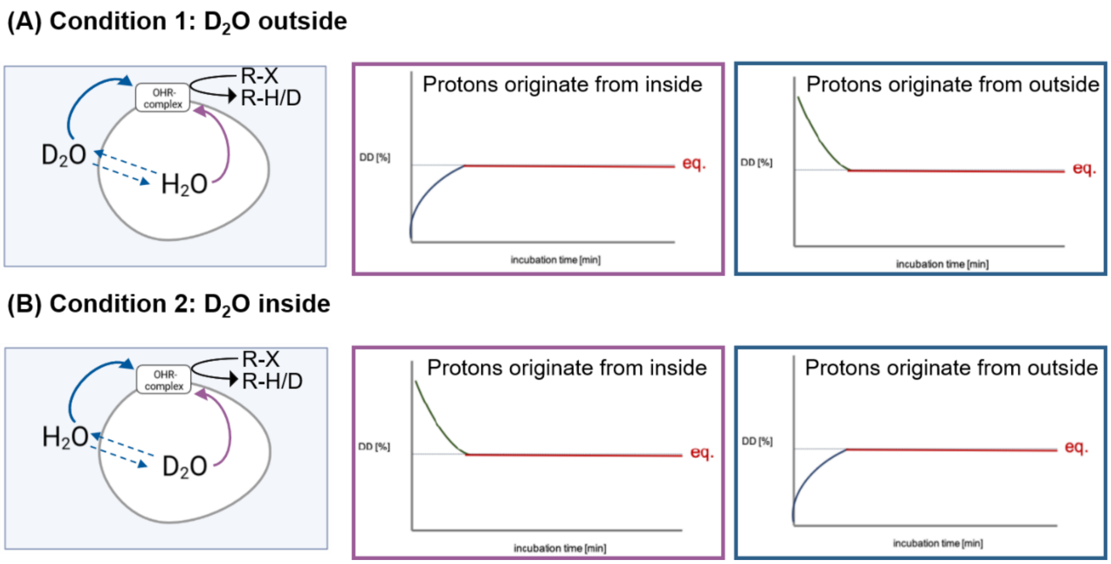

SUPPLEMENTARY FIGURE 1 | The expected progression of deuterium incorporation (DD%) into the formed product is depicted before reaching equilibrium between H_2O and D_2O across the cytosolic membrane. (A) In condition 1, where H_2O was initially present inside the cell and D_2O outside, the deuteration degree of the product is expected to increase over time if protons replacing the halogen atom during dehalogenation originate from the inside (purple framed box). Conversely, if the protons come from outside, the deuteration degree would decrease over time (blue framed box). **(B)** In condition 2, with D_2O inside and H_2O outside the cell at the start of the activity assay, a different scenario is expected. If protons originate from the inside, mainly deuterium is incorporated into the dehalogenation product initially, resulting in a high initial deuteration degree. However, with ongoing incubation, the deuteration degree would gradually decrease and reach an equilibrium (purple framed box). If protons originate from outside, mainly protons are incorporated into the dehalogenation products initially (blue framed box).

1.2 Analysis of the isotope pattern

To analyze the isotope pattern of trichlorobenzene (TCB) and dibromophenol (DBP) formed as products in *in vitro* dehalogenase activity assays, we compared the natural isotope distribution of the molecular ion peak with a labeled molecular ion peak. During the activity assays, deuterium was incorporated into TCB or DBP. Ion abundance was quantified by analyzing the signal area. Extracted Ion Chromatograms (EICs) were generated for m/z = 180 and m/z = 181 for TCB, or m/z = 250 and m/z = 251 for DBP. The incorporation of deuterium into TCB or DBP leads to a shift in their natural isotope patterns. To investigate if the protons incorporated into the product originated from intracellular or extracellular space, the shift of the most abundant isotopic peaks from m/z = 180 to m/z = 181 for TCB, or from m/z = 250 to m/z = 251 for DBP was considered representative. This shift is exemplified for TCB in Supplementary Figure 2. Deuteration degrees (DD1) were calculated to quantify these shifts.

The ratio of *m/z* = 181 to the sum of *m/z* = 180 plus *m/z* = 181 (or the ratio of *m/z* = 251 to the sum of *m/z* = 250 plus *m/z* = 251) represents the share of the mass shift of +1 atomic mass unit (u).

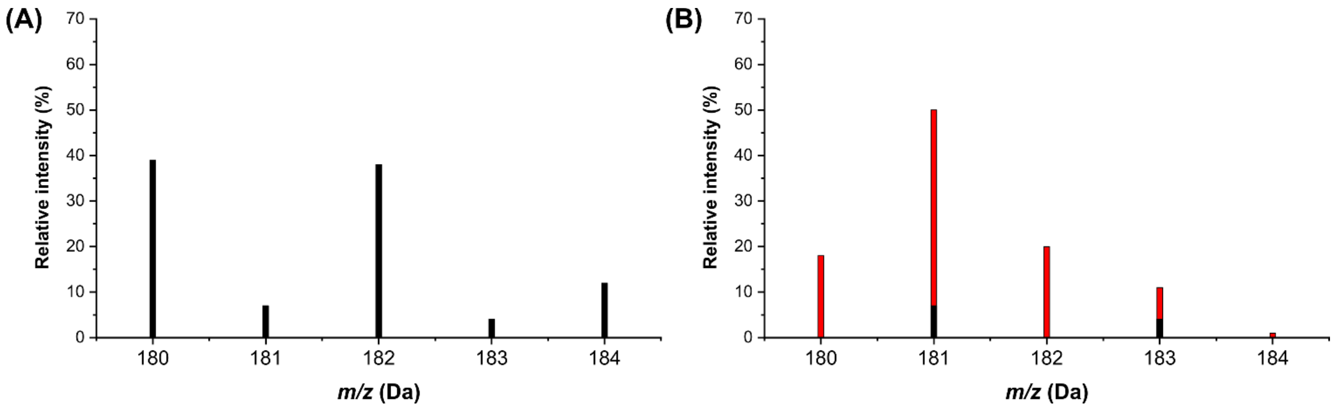

SUPPLEMENTARY FIGURE 2 | Representation of the experimental mass shift resulting from deuterium incorporation into trichlorobenzene (TCB). (A) Isotope pattern of TCB, with the most abundant peaks at *m/z* = 180 and *m/z* = 182, corresponding to the isotopes of chlorine (^{35}Cl and ^{37}Cl) at timepoint t = 15 s. **(B)** Isotope pattern of TCB at timepoint t = 60 min. Deuterium incorporation into TCB causes a mass shift towards *m/z* = 181 and *m/z* = 183.

2 SUPPLEMENTARY DATA

2.1 Supplementary Tables

SUPPLEMENTARY TABLE 1 | Specific activities and turnover numbers per cell were calculated for whole CBDB1 cells (conditions 1 and 2) and crude extracts (conditions 3 and 4) obtained from cultures grown on dibromotyrosine. For conditions 1 and 2, specific activity per cell and turnover rate (k_{cat} cell^{-1}) were significantly higher for incubation times ≤ 1 minute compared to incubation times > 1 min ($p = 0.0051$ or $p = 0.0043$ for condition 1 or 2, and $p = 0.0388$ for condition 3). However, specific activities per cell and k_{cat} cell^{-1} for crude extracts were significantly lower compared to whole cells with $p = 0.0339$ or $p = 0.0104$ for condition 1 or 2 *vs.* condition 3, and $p = 0.0283$ / $p = 0.0087$ for condition 1 / 2 *vs.* condition 4.

Condition: Description	Incubation time	Specific activity (nkat cell^{-1})	k_{cat} (s^{-1} cell^{-1})
**Condition 1:**	**≤ 1 min**	$1.8 \times 10^{-9} \pm 4.3 \times 10^{-10}$	$1.1 \times 10^{6} \pm 2.6 \times 10^{5}$
Whole cells, 5.8×10^{7} cells, D_2O outside	**> 1 min**	$4.3 \times 10^{-10} \pm 1.9 \times 10^{-10}$	$2.6 \times 10^{5} \pm 1.2 \times 10^{5}$
**Condition 2:**	**≤ 1 min**	$1.6 \times 10^{-9} \pm 3.8 \times 10^{-10}$	$9.8 \times 10^{5} \pm 2.3 \times 10^{5}$
Whole cells, 4.2×10^{7} cells, D_2O inside	**> 1 min**	$4.1 \times 10^{-10} \pm 2.8 \times 10^{-10}$	$2.5 \times 10^{5} \pm 1.7 \times 10^{5}$
**Condition 3:**	**≤ 1 min**	$1.3 \times 10^{-10} \pm 2.6 \times 10^{-11}$	$8.0 \times 10^{4} \pm 1.6 \times 10^{4}$
Crude extract of 8.0×10^{8} cells, 20% D_2O	**> 1 min**	$5.1 \times 10^{-11} \pm 2.8 \times 10^{-11}$	$3.1 \times 10^{4} \pm 1.7 \times 10^{4}$
**Condition 4:**	**≤ 1 min**	$8.2 \times 10^{-11} \pm 1.4 \times 10^{-11}$	$4.9 \times 10^{4} \pm 8.4 \times 10^{3}$
Crude extract of 8.0×10^{8} cells, 80% D_2O	**> 1 min**	$4.3 \times 10^{-11} \pm 2.3 \times 10^{-11}$	$2.6 \times 10^{4} \pm 1.4 \times 10^{4}$

2.2 Supplementary Figures

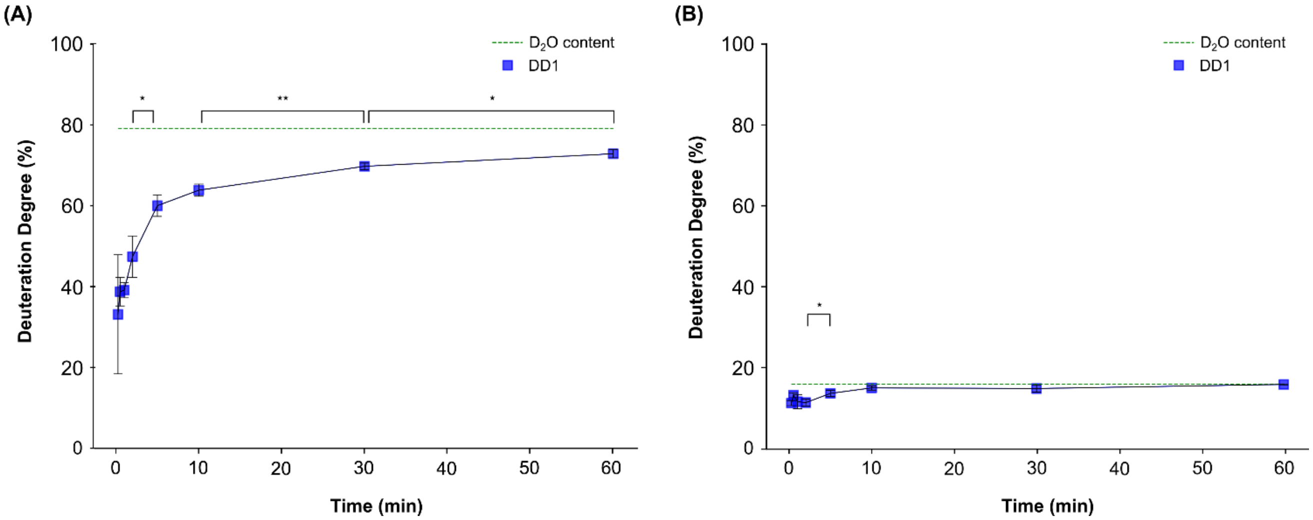

SUPPLEMENTARY FIGURE 3 | Deuteration degree progression in an *in vitro* activity assay with *Dehalococcoides mccartyi* strain CBDB1 cells cultured with 1,2,4,5-tetrabromobenzene. Tetrachlorobenzene was used as the substrate in the activity tests. The measured deuteration degrees of the reaction product, trichlorobenzene, are represented by blue squares at different incubation times. The activity assays were performed in triplicates, and the mean values and standard deviations are displayed. Statistical significance was assessed using a paired two-sample t-test, with significance levels indicated by stars (* $p < 0.05$, ** $p < 0.01$). The calculated deuteration degree (DD1), representing the expected equilibrium deuteration degree, is shown as a green dashed line. **(A)** Deuteration degree progression for condition 1, where initially H_2O was inside the 200 µL cell suspension, and 800 µL of D_2O-based activity master mix was added outside. **(B)** Deuteration degree progression for condition 2, where initially D_2O was inside the cell, and the activity master mix was composed of H_2O.

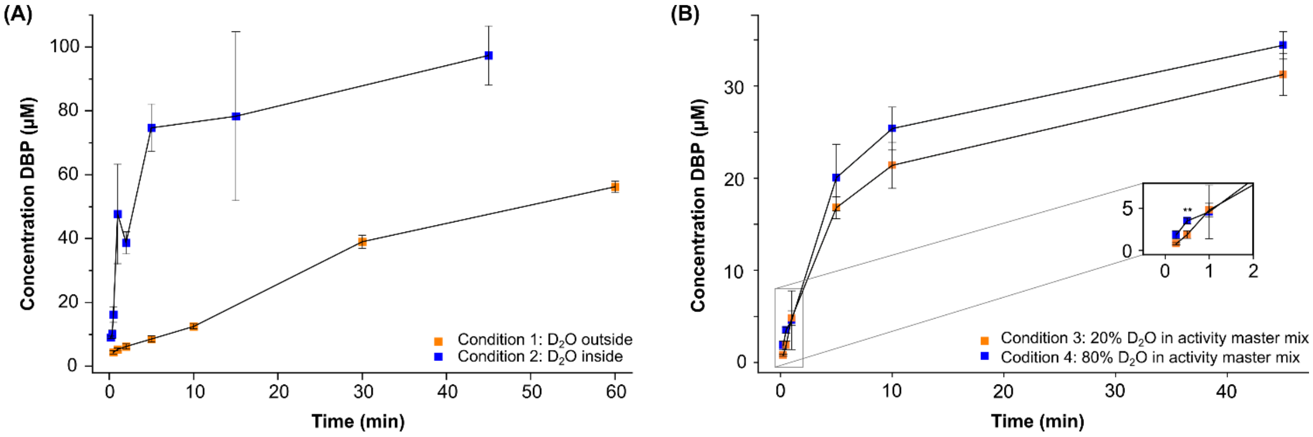

SUPPLEMENTARY FIGURE 4 | Time course of dibromophenol (DBP) product formation in *in vitro* activity assays conducted with whole cells and crude extracts obtained from *Dehalococcoides mccartyi* strain CBDB1 cells cultured on 3,5-dibromo-*L*-tyrosine. The activity assays were performed in triplicates, and the mean values along with standard deviations are presented. Statistical significance was assessed using a paired two-sample t-test, with significance levels indicated by stars (** $p < 0.01$). **(A)** The orange squares represent the DBP product formation over time using CBDB1 cells with H_2O inside and an activity master mix composed of D_2O (condition 1), while the blue squares represent the DBP product formation over time using CBDB1 cells with D_2O inside and an activity master mix composed of H_2O (condition 2). Cells used here were obtained from different growth phases and therefore had different fitness. **(B)** The DBP product formation over time using CBDB1 crude extracts along with an activity master mix containing 20% D_2O (condition 3, blue squares) or an activity master mix containing 80% D_2O (condition 4, orange squares), respectively.

(A)

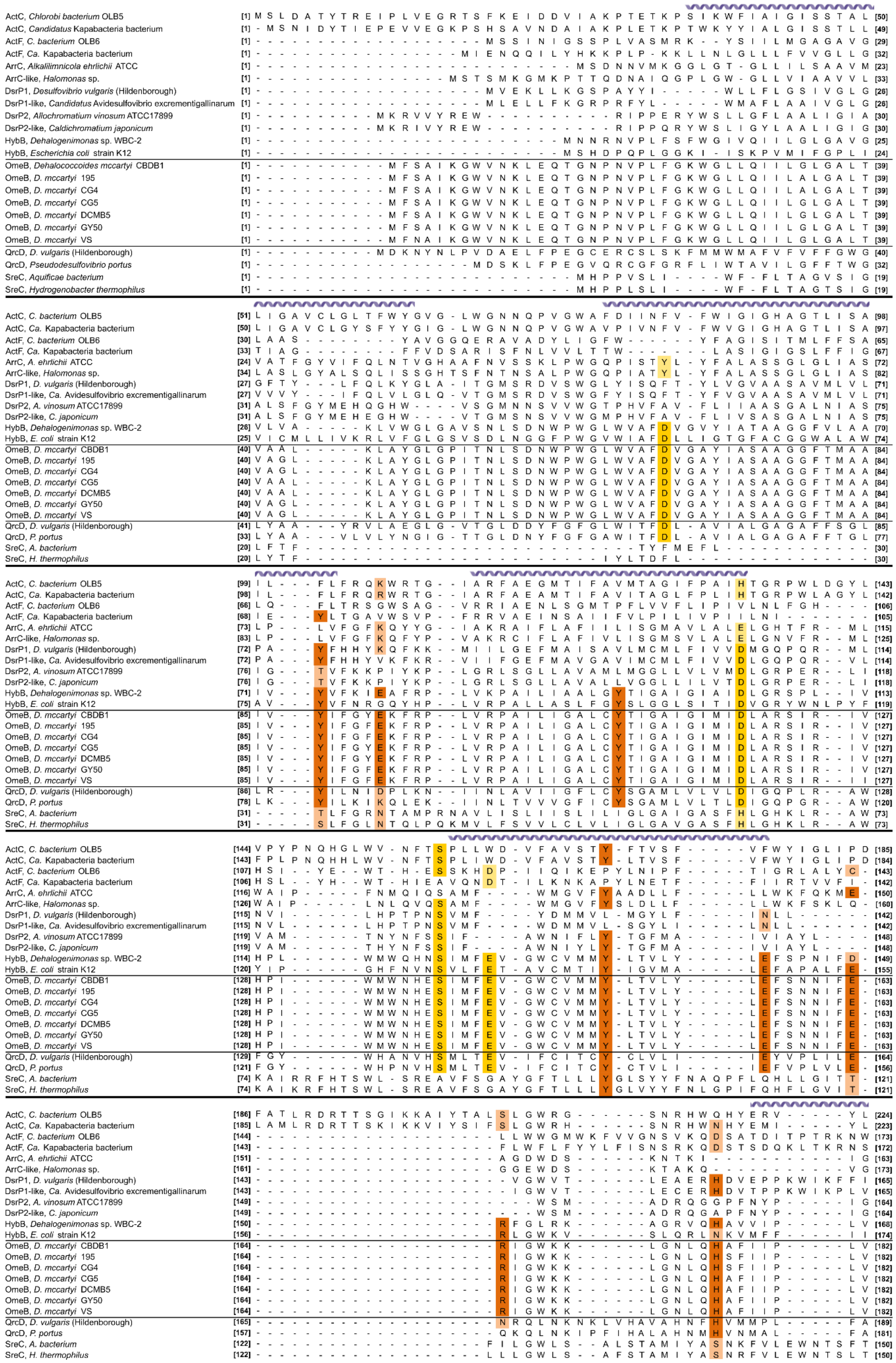

(A)

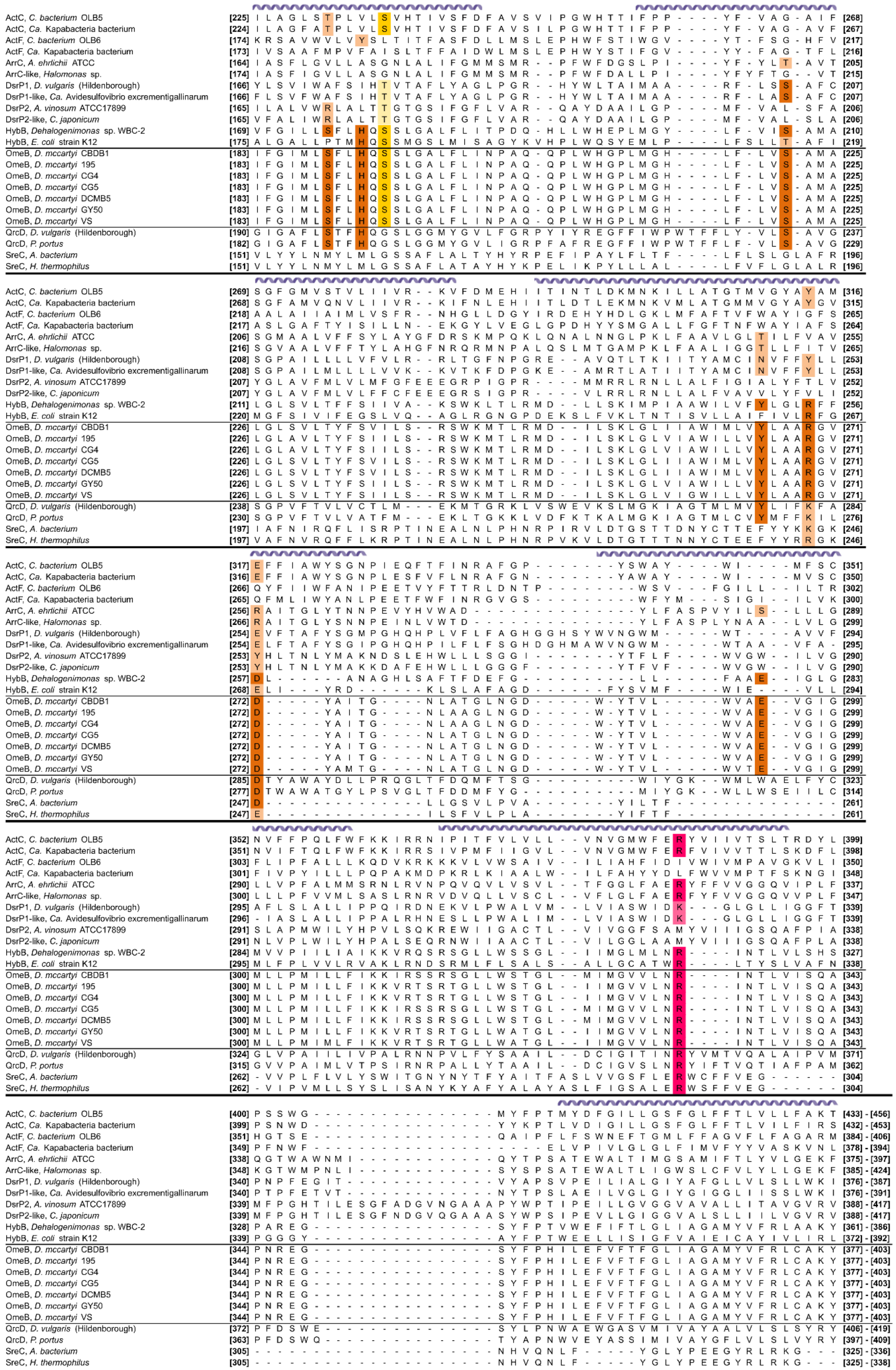

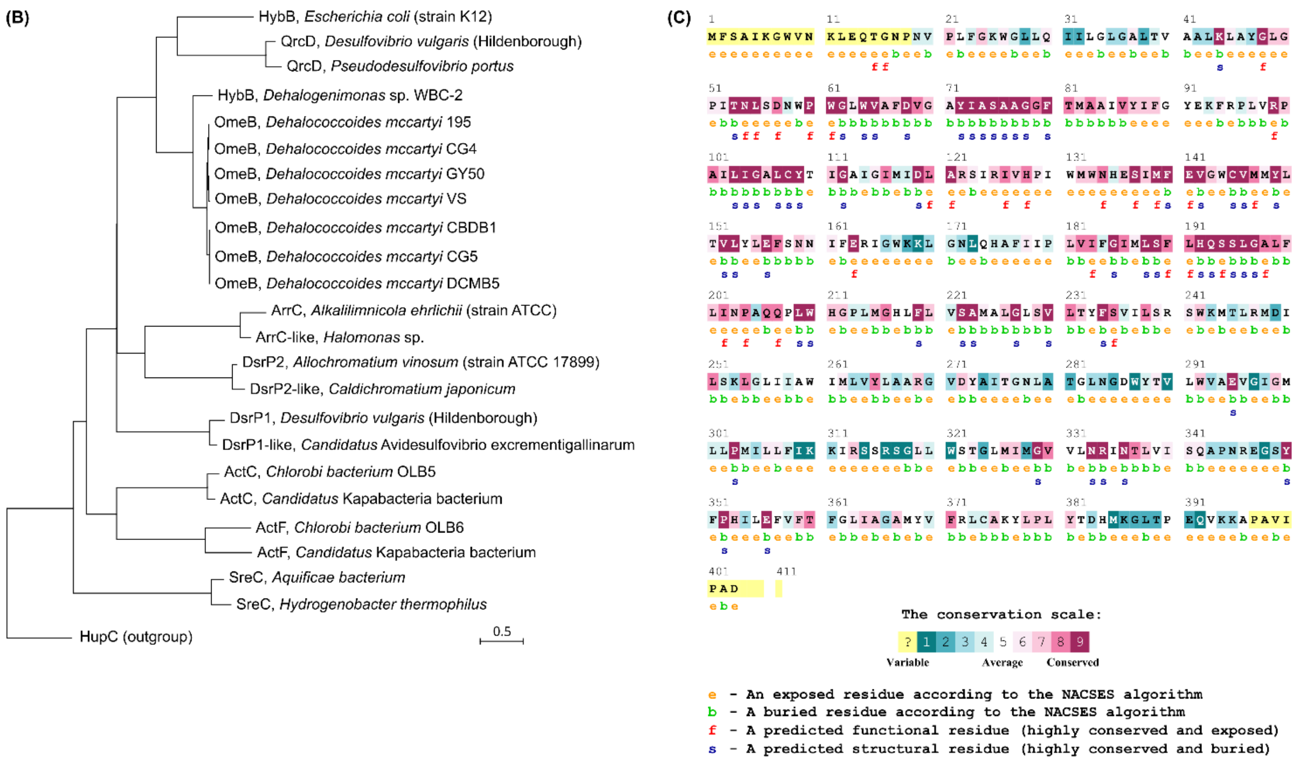

SUPPLEMENTARY FIGURE 5 | Analysis of the evolutionary relationship among various NrfD-like proteins, including the OmeB subunit of the organohalide respiratory complex in *Dehalococcoides mccartyi* strain CBDB1. (A) Multiple sequence alignment (MSA) of 23 NrfD homologous amino acid sequences. Conserved amino acid residues mostly predicted to be involved in proton transport and forming two putative half-channels are highlighted in orange. These half-channels converge at a highly conserved arginine residue (highlighted in pink). Conserved amino acid predicted to be involved in forming a putative quinone-binding site are highlighted in ochre. The purple spiral at the top represents the α-helix positions in OmeB. **(B)** Phylogenetic analysis of the 23 NrfD homologs using the Maximum Likelihood method, with evolutionary distances calculated using Poisson correction. OmeB homologs are grouped with other NrfD-like proteins, with QrcD and HybB subunits being the closest relatives to OmeB. HupC was selected as an outgroup. **(C)** The ConSurf server was used to analyze the evolutionary conservation and identify critical sites within the amino acid sequences of 79 NrfD homologous proteins. Highly conserved amino acids are shown in purple and are located in the N-terminal transmembrane helices (TMHs) 2-5, while C-terminal TMHs show lower conservation (colored cyan).

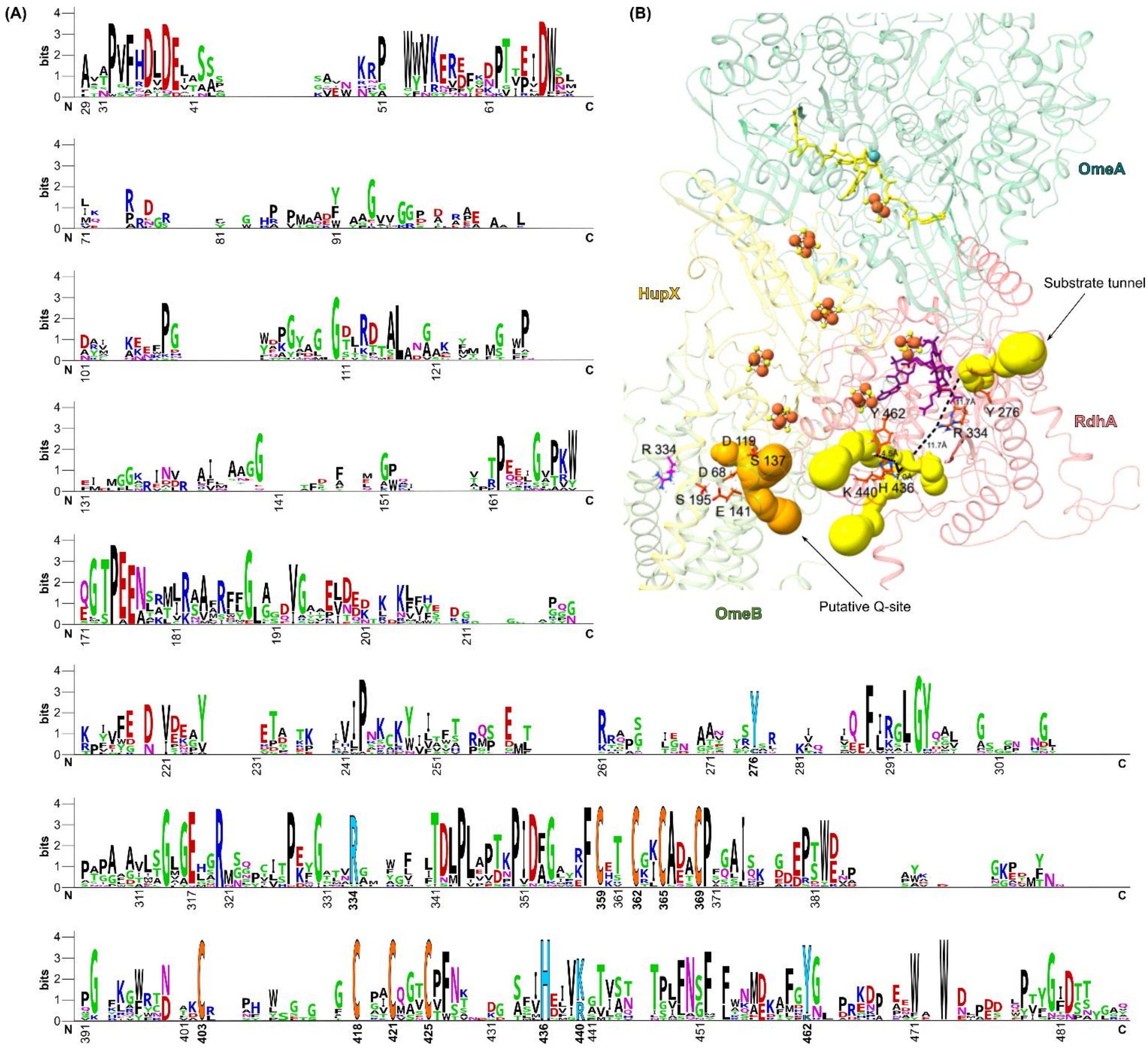

SUPPLEMENTARY FIGURE 6 | Bioinformatic analysis of reductive dehalogenases (RdhA) in *Dehalococcoides mccartyi* strain CBDB1. (A) Sequence logo from multiple sequence alignment of 32 putative RdhA homologous proteins in *D. mccartyi* strain CBDB1, excluding the N-terminal TAT leader signal peptide. The logo highlights the highly conserved [4Fe-4S] cluster binding motifs $Cx_2Cx_2Cx_3C$ and $Cx_nCx_2Cx_3C$ (orange) and the conserved amino acid residues that may form continuous proton conducting paths within RdhA (light-blue). **(B)** The OmeAB-HupX-RdhA submodule of the organohalide respiratory complex in strain CBDB1 is shown in cartoon conformation, with conserved amino acids and cofactors. The OmeAB-HupX submodule and CbrA (RdhA encoded by cbcbA84 locus) were modeled using AlphaFold2. The OmeA subunit (dark green) is predicted to contain a [3Fe-4S] cluster and a *bis*-MGD cofactor (yellow, stick conformation), while HupX subunit (yellow) would bind four [4Fe-4S] clusters arranged in an electron-conducting ‘wire’. In the model, its distal [4Fe-4S] cluster is in proximity of ca. 10 Å to the putative Q-site in the OmeB subunit (light green). The CbrA subunit (pink) contains a cobalamin cofactor (purple, stick conformation) and two [4Fe-4S] clusters, with an accessible active site through a water-filled substrate tunnel (yellow bubble). Distances in Ångström between conserved amino acid residues that might form a continuous proton conducting path within RdhA are shown. Conserved amino acids are displayed in orange or pink stick representation, and putative water-filled cavities in RdhA or OmeB are shown in yellow or orange, respectively.

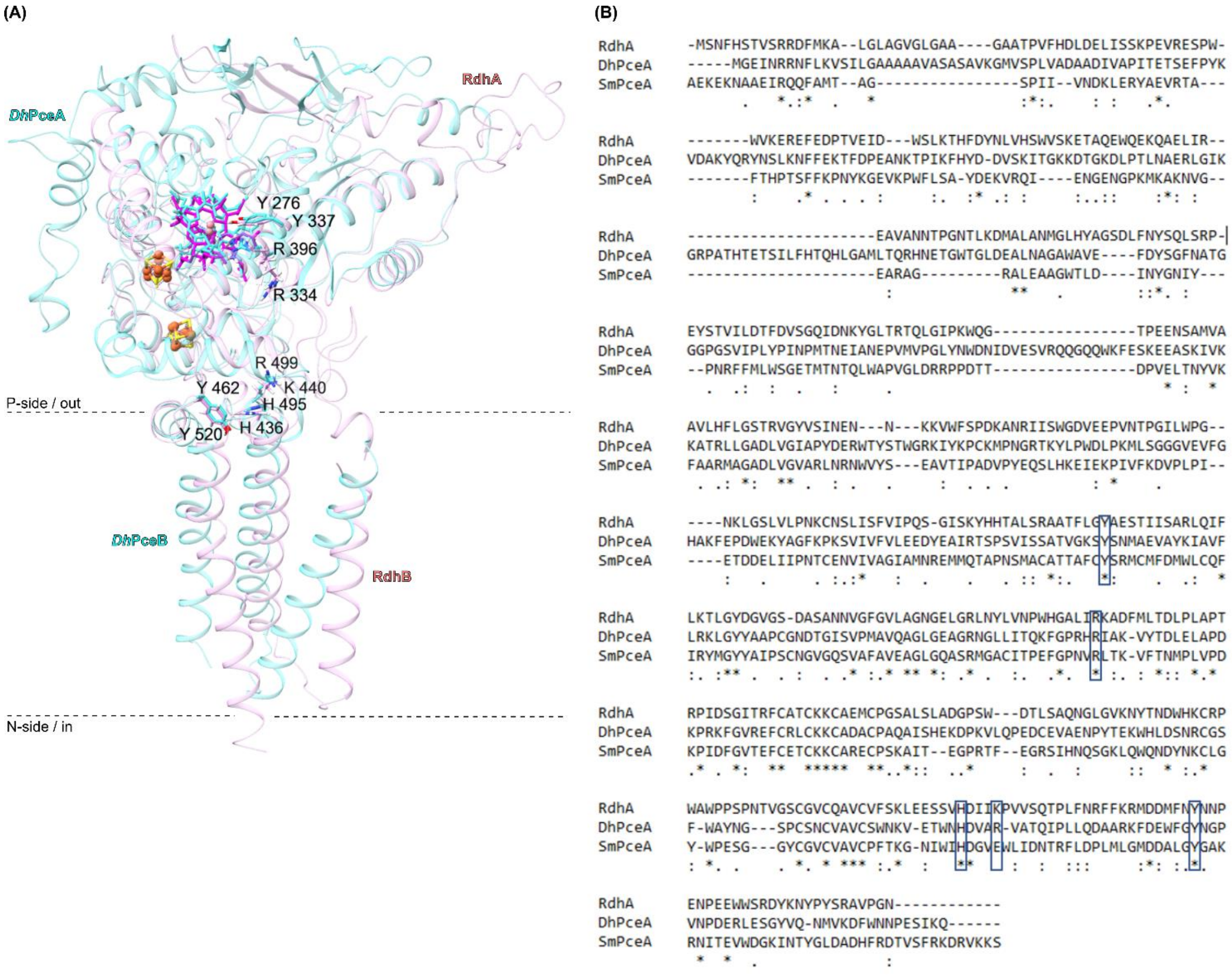

SUPPLEMENTARY FIGURE 7 | Homology-based models of RdhAB proteins from the organohalide respiratory (OHR) complex in *Dehalococcoides mccartyi* strain CBDB1 were superimposed with the experimental cryo-EM structure of *Dh*PceAB from *Desulfitobacterium hafniense* (Cimmino et al., 2023). (A) The RdhAB submodule (loci cbdbA84 and cbdbA85) of the OHR complex in strain CBDB1, computed using AlphaFold2, is depicted in a cartoon conformation (pink). RdhAB was superimposed with the experimental structure of *Dh*PceAB (light blue cartoon conformation) obtained through cryo-EM. Both RdhA (pink) and *Dh*PceA subunits contain a cobalamin cofactor (purple or blue, stick conformation) and two [4Fe-4S] clusters. Conserved amino acids in RhdA/DhPceA (Y276/Y337, R334/R396, H436/495, K440/R499, and Y462/Y520) are shown in light blue (for DhPceAB) or pink (for RdhAB) stick representation. **(B)** Multiple sequence alignment of RdhA from *D. mccartyi* strain CBDB1, PceA from *D. hafniense*, and PceA from *Sulfurospirillum multivorans*. Conserved amino acid residues, mostly predicted to be involved in proton transport towards the reductive dehalogenase's active site, as highlighted in (A), are marked. Multiple sequence alignment was conducted using MUSCLE algorithm integrated in MEGA 11.

3 SUPPLEMENTARY DISCUSSION

3.1 The OHR complex discriminates against deuterium translocation across the membrane

In our experiments the deuteration degree in the product never reaches the preset assay deuterium content (Figure 3A, Supplementary Figure 3A), even when working with crude extracts (Figure 4B), where no difference in deuterium content should occur between the cytoplasm and the exterior. This indicates that the OHR complex strongly discriminates against the uptake of deuterium for product protonation.

It has been frequently reported that proton/deuterium channeling through membrane proteins is limited because of strong discrimination against deuterium, resulting in a significantly lower conductivity of deuterium through proteins. Due to the simple isotope mass effect (deuterium ions have twice the mass of protons) deuterium has approximately 1.5 times lower conductivity in liquids composed of D_2O than protons in H_2O liquids (Lewis and Doody, 1933; Noel et al., 1974). However, when protein residues are involved even lower conductivity has been observed, originating from a kinetic isotope effect that is greater than the simple isotope mass effect observed in solvents (Agmon, 1995). For transmembrane proteins kinetic isotope effects of 2.5 (Hallen and Nilsson, 1992) and 5-7 have been determined (Karpefors et al., 1999; Salomonsson et al., 2008), which significantly affects proton tunneling through membrane proteins that is mainly achieved through the hydrogen-bonded chain mechanism, an extension of the Grotthuss-type mechanism (Agmon, 1995). In this mechanism, water molecules or amino acid residues capable of establishing hydrogen bonds conduct protons through a 'hop-turn' mechanism, which is significantly faster than a simple hydrodynamic diffusion of individual H_3O^+ molecules (Nagle and Morowitz, 1978; Nagle and Tristram-Nagle, 1983). Even in the case of water-filled protein channels, protons do not simply pass through the tunnel. They instead interact with the amino acids within the tunnel, contributing to a high kinetic isotope effect (DeCoursey and Cherny, 1997; Mould et al., 2000; DeCoursey, 2003). After a single proton conduction event, rearrangement of the hydrogen-bonded water or amino acid network is required, before another proton can be transferred (Conway et al., 1956). The introduction of deuterium into this 'proton wire' hinders this mechanism, making it more difficult than free Grotthuss-type proton transfer in solvents.

In our experiments, we observed a significant kinetic isotope effect under condition 2, leading to affected incorporation of deuterium. Our observations are consistent with previous studies on

deuterium isotope effects in proton channels (Hallen and Nilsson, 1992; Karpefors et al., 1999; Salomonsson et al., 2008). The strong discrimination against deuterium in the OHR complex is not occurring during the organohalide reduction and subsequent protonation at the active site of the RdhA. Instead, the fractionation likely occurs during the transport of protons/deuterium through the OHR proteins from the cytoplasmic side towards the periplasmic active site in RdhA. Once the proton or deuterium is shuttled, it is readily incorporated into the dehalogenation product by the action of RdhA. Thus, the discrepancies between the theoretical and the observed deuteration degree under conditions 1 and 4 can be attributed to physicochemical differences between H_2O and D_2O.

4 REFERENCES

Agmon, N. (1995). The grotthuss mechanism. *Chem Phys Lett* 244, 456–462.

Conway, B. E., Bockris, J. O., and Linton, H. (1956). Proton conductance and the existence of the H_3O^+ ion. *J Chem Phys* 24, 834–850.

DeCoursey, T. E. (2003). Voltage-gated proton channels and other proton transfer pathways. *Physiol Rev* 83, 475–579.

DeCoursey, T. E., and Cherny, V. V. (1997). Deuterium isotope effects on permeation and gating of proton channels in rat alveolar epithelium. *J Gen Physiol* 109, 415–434.

Hallen, S., and Nilsson, T. (1992). Proton transfer during the reaction between fully reduced cytochrome *c* oxidase and dioxygen: pH and deuterium isotope effects. *Biochemistry* 31, 11853–11859.

Karpefors, M., Ädelroth, P., Aagaard, A., Smirnova, I. A., and Brzezinski, P. (1999). The deuterium isotope effect as a tool to investigate enzyme catalysis: proton-transfer control mechanisms in cytochrome *c* oxidase. *Isr J Chem* 39, 427–437.

Lewis, G. N., and Doody, T. C. (1933). The mobility of ions in H^2H_2O. *J Am Chem Soc* 55, 3504–3506.

Mould, J. A., Li, H.-C., Dudlak, C. S., Lear, J. D., Pekosz, A., Lamb, R. A., et al. (2000). Mechanism for proton conduction of the M_2 ion channel of influenza A virus. *J Biol Chem* 275, 8592–8599.

Nagle, J. F., and Morowitz, H. (1978). Molecular mechanisms for proton transport in membranes. *Proc Natl Acad Sci U.S.A.* 75, 298–302.

Nagle, J. F., and Tristram-Nagle, S. (1983). Hydrogen bonded chain mechanisms for proton conduction and proton pumping. *J Membr Biol* 74, 1–14.

Noel, K., Roberts, K., Helen, L., and Northey, L. (1974). Proton and deuteron mobility in normal and heavy water solutions of electrolytes. *J Am Chem Soc* 70, 253–262.

Salomonsson, L., Brändén, G., and Brzezinski, P. (2008). Deuterium isotope effect of proton pumping in cytochrome *c* oxidase. *Biochim Biophys Acta* 1777, 343–350.
